# Aerobic Bioaugmentation to Decrease Polychlorinated Biphenyl (PCB) Emissions from Contaminated Sediments to Air

**DOI:** 10.1021/acs.est.2c01043

**Published:** 2022-09-30

**Authors:** Christian M. Bako, Andres Martinez, Jessica M. Ewald, Jason B. X. Hua, David J. Ramotowski, Qin Dong, Jerald L. Schnoor, Timothy E. Mattes

**Affiliations:** † The Department of Civil & Environmental Engineering, 4105 Seamans Center for the Engineering Arts & Sciences, University of Iowa, Iowa City, Iowa 52245, United States; ‡ IIHR—Hydroscience & Engineering, University of Iowa, Iowa City, Iowa 52242, United States

**Keywords:** polychlorinated biphenyls, contaminant fate and transport, Paraburkholderia xenovorans LB400, passive sampling, bioremediation, biodegradation, biosurfactants, bioavailability

## Abstract

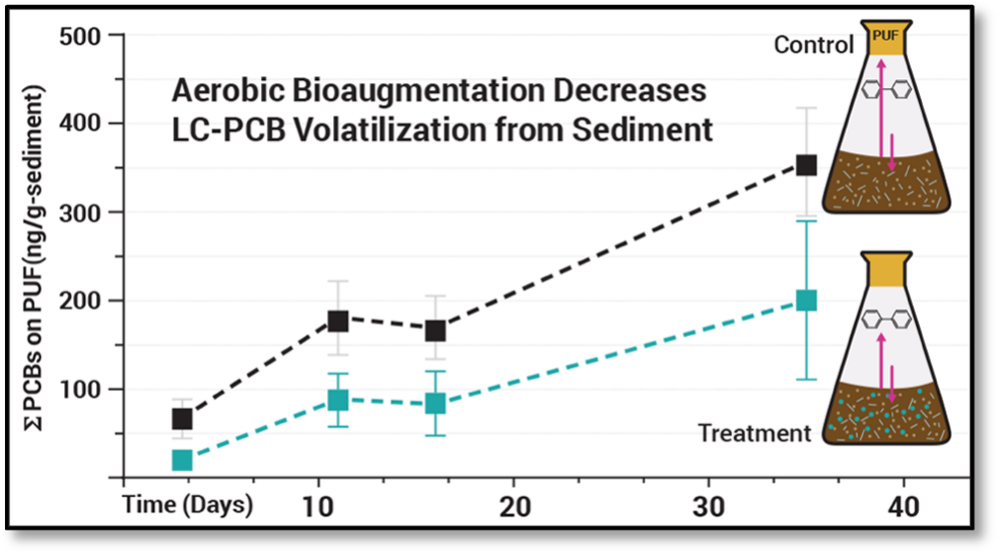

We conducted experiments to determine whether bioaugmentation with aerobic, polychlorinated biphenyl (PCB)-degrading microorganisms can mitigate polychlorinated biphenyl (PCB) emissions from contaminated sediment to air. *Paraburkholderia xenovorans* strain LB400 was added to bioreactors containing PCB-contaminated site sediment. PCB mass in both the headspace and aqueous bioreactor compartments was measured using passive samplers over 35 days. Time-series measurements of all 209 PCB congeners revealed a 57% decrease in total PCB mass accumulated in the vapor phase of bioaugmented treatments relative to non-bioaugmented controls, on average. A comparative congener-specific analysis revealed preferential biodegradation of lower-chlorinated PCBs (LC-PCBs) by LB400. Release of the most abundant congener (PCB 4 [2,2′-dichlorobiphenyl]) decreased by over 90%. Simulations with a PCB reactive transport model closely aligned with experimental observations. We also evaluated the effect of the phytogenic biosurfactant, saponin, on PCB bioavailability and biodegradation by LB400. Time-series qPCR measurements of biphenyl dioxygenase (*bphA*) genes showed that saponin better maintained *bphA* abundance, compared to the saponin-free treatment. These findings indicate that an active population of bioaugmented, aerobic PCB-degrading microorganisms can effectively lower PCB emissions and may therefore contribute to minimizing PCB inhalation exposure in communities surrounding PCB-contaminated sites.

## Abstract 2

Beneficial microbes can be added to the sediments of PCB-contaminated water bodies to reduce the movement of pollutants to air and to potentially protect humans from inhalation exposure.

## Introduction

Polychlorinated biphenyls (PCBs) remain ubiquitous environmental pollutants decades after sales were banned in the United States in 1979 and globally in 2004.^1^ Inhalation of airborne PCBs could be a significant route of cumulative human exposure for certain vulnerable populations due to emissions from modern, unregulated sources of PCBs^2^ and legacy sources, such as PCB-contaminated Superfund sites.^3−12^ This work focuses on legacy sediment sources. A study found that infants born near New Bedford Harbor during dredging operations had consistently higher PCB levels in umbilical cord serum than infants born after dredging.^13^ Their findings not only suggest that dredging operations directly contributed to higher levels of human exposure to airborne PCBs but also that the site was a significant source of PCBs to the surrounding community before dredging. This is alarming because even low-level prenatal PCB exposure is associated with lower birth weights, endocrine system disruption, and cognitive impairments in children that may not be evident until school age.^14−16^ Furthermore, an analysis of hospitalization data from PCB-contaminated areas in New York State supported the hypothesis that chronic exposure to volatile PCBs increases the likelihood of developing cardiovascular disease, hypertension, and diabetes.^17^ This hypothesis is further supported by data showing that thyroid-related health risks of airborne polychlorinated biphenyls are highest in communities living closest to the PCB-contaminated New Bedford Harbor.^18^

PCB congeners most likely to volatilize from contaminated sediments are the “lightly chlorinated” (LC)-PCBs. The “highly chlorinated” (HC)-PCBs are less soluble, less volatile, and therefore less mobile in the environment. Historic anaerobic reductive dechlorination of HC-PCBs generates LC-PCBs that are more likely to volatilize than their parent congeners.^19−21^ Therefore, PCB-contaminated sites where reductive dechlorination is ongoing may contribute to continuous, low-level emissions of LC-PCB transformation products that can be significantly elevated immediately following sediment perturbation or atmospheric exposure in tidal systems, dredging operations, or drought.^22^ We propose that some LC-PCB emissions can be mitigated through bioaugmentation of aerobic microorganisms which have naturally evolved the ability to oxidize PCB congeners through the upper *bph* pathway.^23,24^

*Paraburkholderia xenovorans* strain LB400, isolated from PCB-contaminated landfill soil in 1985^25−27^ and widely regarded as an efficient *bph* pathway utilizer,^28−32^ is the bioaugmentation strain used here. Our previous work reproduced the results of these early PCB biodegradation assays using modern analytical instrumentation and showed how LB400 preferentially biodegraded bioavailable LC-PCBs but struggled to biodegrade LC-PCBs bound to sediment particles gathered from a contaminated site.^33−35^ That experiment was designed to demonstrate that limited mass transfer (low bioavailability) is a key constraint to effective PCB biodegradation and to document LB400′s broad congener specificity.^36−38^ Compounds present in plant rhizospheres can act as biosurfactants to make PCBs and other hydrophobic organic compounds tightly bound to organic matter more bioavailable for microbial transformation.^39^ For example, the phytogenic biosurfactant, saponin, can aid aerobic PCB-contaminated sediment remediation.^40,41^ Combined anaerobic–aerobic bioaugmentation by use of bioamended activated carbon is a promising alternative for stimulating PCB biodegradation processes in situ.^42−44^ However, bioaugmentation strategies for mitigating airborne PCB emissions using aerobic PCB-degrading microorganisms have not been documented.

A National Research Council report on sediment dredging at Superfund Megasites called for development of improved methods to monitor and reduce contaminant releases before, during, and after dredging operations to better predict and prevent human exposure.^9^ Our work uses kinetic phase passive sampling measurements to test the hypothesis that atmospheric PCB emissions can be minimized through targeted aerobic bioremediation of specific congeners most likely to volatilize from legacy sources.^3,45−47^ We measured volatile PCB accumulation on polyurethane foam (PUF) passive samplers deployed in lab-scale bioreactors containing sediment slurry and compared results of bioaugmented treatments to non-bioaugmented treatments over a period of 35 days. We also investigated whether the phytogenic biosurfactant, saponin, improved PCB bioavailability in aqueous bioreactor compartments by measuring freely dissolved PCBs with solid-phase microextraction (SPME) fiber passive samplers.^48−53^ We evaluated our measurements with a three-way mixed-effects statistical analysis and output from a PCB reactive transport model optimized using parameters unique to this study.

## Materials and Methods

### Sediment and Site Description

**Figure 1:**
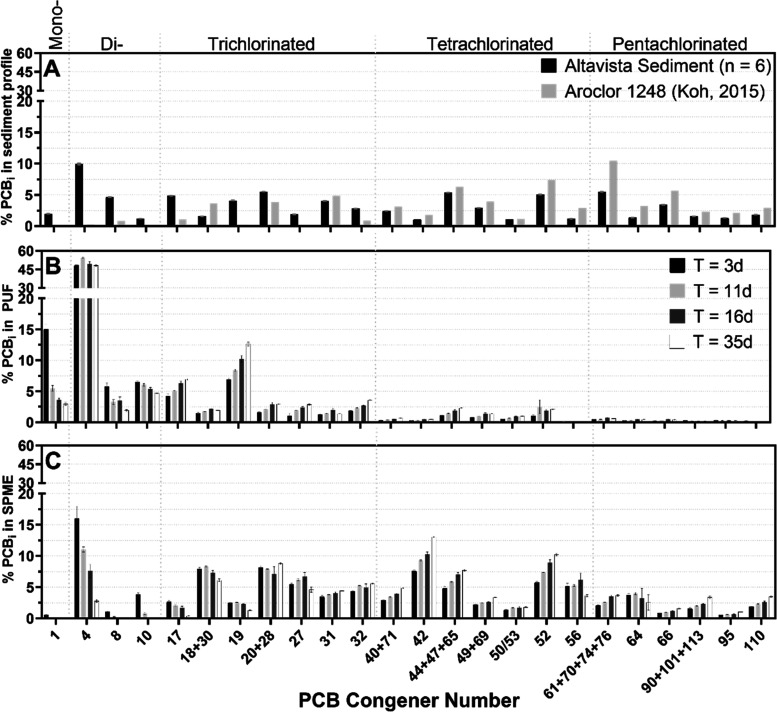
(A) PCB congener profile of Altavista sediment (previously determined).^33^ For ease of reference, a condensed profile of individual PCB congeners is shown along the horizontal axis although all 209 congeners (minus surrogate and internal standards) were measured. The condensed profile represents congeners ≥2% of the total profile, by mass fraction, in either PUF or SPME samples at any time point in any treatment. (B) PCB congener profile detected in the headspace of bioreactors using PUF passive samplers. Values shown are from the saponin-free sediment control group. Mass fractions of mono- and dichlorinated congeners trend lower or remain level as the relative contribution of more slowly desorbing tri-, tetra-, and pentachlorobiphenyls trends higher with time. (C) Congener profile of freely dissolved PCBs detected in the aqueous compartment of the bioreactors using SPME passive samplers. *T* = 3 days samples capture LC-PCBs that rapidly desorbed from sediment before volatilizing to air whereas later samples capture the more slowly desorbing tri-, tetra-, and pentachlorobiphenyls that are still entering solution or have reached dynamic equilibrium. The error bars represent the standard error of triplicate measurements (*n* = 3; except where otherwise indicated).

Sediment was collected from a decommissioned, PCB-contaminated emergency overflow lagoon located in Altavista, VA (Figure S1). Information on sediment properties (Table S1), homogenization method, and sediment sampling strategy have been previously described.^33^ The average total PCB concentration (∑PCB = sum of 171 congeners) was 6350 ng/g (STD = 312 ng/g, *n* = 6). The PCB congener profile of Altavista sediment most closely resembled the commercial mixture Aroclor 1248 (Figure 1A).^54^ Sediment was not sterilized before use in this experiment.^55−57^

### Bacterial Strain and Growth Conditions

*P. xenovorans*LB400 was grown aerobically at room temperature on a platform shaker in 4 L Erlenmeyer flasks containing 2 L K1 medium^33^ and solid biphenyl crystals (10 mM; 1.55 g/L) as a sole carbon and energy source until the mid-exponential phase (OD_600_ = 1.0). Cells were harvested in 1 L batches by three rounds of centrifugation (5000*g*, 20 min per round) and washed twice with sterile 20 mM phosphate buffered saline solution and resuspended in K1 medium overnight on a platform shaker. The final combined and homogenized cell suspension in K1 medium was used as the bioaugmentation solution (final OD_600_ = 1.2). Using a Pierce BCA Protein Assay Kit (Thermo Scientific; Rockford, IL), the estimated biomass concentration of the bioaugmentation solution was 230 μg/mL as protein. Reactors were bioaugmented with cells (66.7 mL) using a serological pipette.^58^

### Bioreactor Setup

Bioaugmentation experiments included two non-bioaugmented sediment control groups (referred to as “Control” and “Control + Sap”) and two LB400-bioaugmented sediment treatment groups (“LB400” and “LB400 + Sap”). The two sediment control groups (*n* = 3 replicates per timepoint) were saponin-amended bioreactors and saponin-free bioreactors. The treatment groups (*n* = 4 replicates per timepoint) were identical to respective controls except they were bioaugmented with LB400. Passive samplers were used to measure PCB mass in the vapor and aqueous phases at four time points spanning 35 days (*T*_1_ = 3 days, *T*_2_ = 11 days, *T*_3_ = 16 days, and *T*_4_ = 35 days; experimental design matrix shown in Figure S2).

Bioreactors were 250 mL Erlenmeyer flasks containing a PCB-contaminated sediment slurry (10% mass to volume ratio; ∼10 g weathered sediment [wet weight] to 100 mL aqueous solution; determined gravimetrically; Figure S3). For appropriate comparison across control and treatment groups, liquid K1 medium^33^ was used in the saponin-free control because LB400 cells added to the treatment groups were suspended in K1 medium. Sediment-free controls containing LB400 in K1 medium with and without saponin were also established for comparison in qPCR experiments. Saponin salt (Fisher Scientific, Hampton, NH) dissolved in sterile K1 medium was added to +Sap bioreactors at a final concentration of 500 mg/L. This concentration was chosen such that sediment slurry would be conservatively above the critical micelle concentration (CMC) at the average unadjusted bioreactor pH of 6.5.^40,59^ Mouths of established bioreactors were covered with aluminum foil to prevent volatilization of the lowest-molecular-weight congeners from PUF stoppers (Figure S3). Bioreactors were placed on a platform shaker at 150 rpm to accelerate PCB desorption such that accumulation was observable on passive samplers over the experimental timescale. All bioreactors were sampled sacrificially.

### *bphA* Abundance Estimation with qPCR

Sediment slurry (5 mL) and liquid (1.8 mL) samples for nucleic acid extraction were collected from the bioaugmented treatments and the sediment-free controls, respectively, at each sampling point. DNA was extracted from slurry samples with the DNeasy PowerSoil Pro Kit (Qiagen, MD), after centrifugation (20 min; 5000*g*) and decanting of supernatant. DNA was extracted from sediment-free samples with the DNeasy Powerlyzer Microbial kit (Qiagen, MD). DNA concentrations were measured with the Qubit dsDNA high sensitivity assay kit and the Qubit 4 fluorometer (ThermoFisher Scientific, Waltham, MA). The *bphA* gene abundance was measured with qPCR with an ABI 7000 Sequence Detection System (Applied Biosystems, Grand Island, NY) as described previously.^58^ Briefly, each reaction (20 μL) contained 10 μL of Power SYBR Green PCR Master Mix (Invitrogen, Carlsbad, CA), 0.3 μM of forward and reverse *bphA* primers (Table S2), and 0.1 μL of bovine serum albumin (20 mg/mL; New England Biolabs, Ipswich, MA). A standard curve using known amounts of *bphA* cloned into the 2.1-TOPO vector, prepared in triplicate was used to quantify *bphA* abundance. Melt curve analysis revealed single peaks in both the standards and samples at a temperature of 86.3 °C. Additional primer and QA/QC details that satisfy MIQE guidelines^60^ are in Tables S2 and S3.

### Passive Sampling

Passive samplers measured the PCB congener mass in the gas and aqueous phases of each bioreactor. PUF cylinders (height ≈ 1 in. (2.5 cm) × radius = 0.75 in. (1.9 cm); Tisch Environmental Inc., OH), placed in the neck of each flask and subsequently covered with aluminum foil (Figure S3), were used to measure PCB congeners that volatilized into the bioreactor headspace.^61,62^ SPME fibers coated with a 10 μm thick layer of poly(dimethylsiloxane) (PDMS) were immersed in sediment slurry to measure freely dissolved PCB congeners.^63,64^ SPME fibers were deployed as ∼2.5 cm segments totaling 30–40 cm per bioreactor. The PDMS volume was 6.9 × 10^–8^ L/cm-fiber. At the time of sampling, PUF samplers were removed from bioreactors, wrapped in clean aluminum foil, and stored at −10 °C until PCB extraction and analysis.

SPME fibers were retrieved from bioreactors using tweezers as sediment slurry was transferred from the flask to an evaporating dish inside a fume hood. Recovered SPME fiber segments were rinsed with Optima water, wiped dry with a Kimwipe, measured with a ruler, placed in glass GC-vial inserts filled with hexane to facilitate complete PCB desorption from the fiber’s PDMS coating, and spiked with internal standard compounds as described in PCB Extraction Methods.

### PCB Extraction Methods

PCBs were extracted from PUF via pressurized liquid extraction (PLE) with equal parts of acetone and hexane using an accelerated solvent extractor (ASE). Before PLE, the ASE cell containing the PUF sampler was spiked with surrogate standards PCB 14 (50.81 ng; 3,5-dichlorobiphenyl), deuterated PCB 65-d5 (52.5 ng; 2,3,5,6-tetrachlorobiphenyl-*d*_5_, deuterated), and PCB 166 (52.56 ng; 2,3,4,4′,5,6-hexachlorobiphenyl; Cambridge Isotope Laboratories, Inc.). The sample extract resulting from PLE was concentrated within a TurboVap collection vial to ∼1 mL using a TurboVap II Concentration Workstation (Biotage, Uppsala, Sweden). The final hexane extract was passed through a Pasteur pipette filled with 0.1 g of combusted silica gel and 1 g of acidified silica gel (2:1 silica gel/sulfuric acid by weight) and eluted with ∼10 mL of hexane.^65^ Samples were again concentrated to ∼1 mL and transferred to a gas chromatography vial. The final sample was spiked with PCB 204 (19.6 ng; 2,2′,3,4,4′,5,6,6′-octachlorobiphenyl; Cambridge Isotope Laboratories, Inc.) as internal standard.

### PCB Quantification

GC-MS/MS (Agilent 7890A GC system, Agilent 7000 Triple Quad, Agilent 7693 autosampler) in multiple reaction monitoring mode (MRM) was used for identification and quantification of 209 PCBs as 171 chromatographic peaks. The GC was equipped with a Supelco SPB-Octyl capillary column (50% *n*-octyl, 50% methyl siloxane, 30 m × 0.25 mm ID, 0.25 μm film thickness) with helium as carrier gas flowing at 0.75 mL/min and nitrogen as collision gas. The GC operated in solvent vent injection mode at the following injection conditions: initial temperature 45 °C, initial time 0.06 min, ramp 600 °C/min to inlet temperature 325 °C at 4.4 psi. The GC oven temperature program was 45 °C for 2 min, 45–75 °C at 100 °C/min and hold for 5 min, 75–150 °C at 15 °C/min and hold for 1 min, and 150–280 °C at 2.5 °C/min and finally hold for 5 min (total run time 70.86 min). The triple quadrupole MS electron ionization source was set to 260 °C. Additional details can be found in the accompanying dataset deposited in the Iowa Research Online data repository.^66^

### PCB Reactive Transport Modeling

The reactive transport model developed for this study^67^ consisted of three major components: (1) PCB sorption–desorption processes from suspended particles in the aqueous phase, (2) biotransformation in the aqueous phase, and (3) air–water exchange. Sorption–desorption processes were assumed to be at chemical equilibrium, and the concentration of suspended particles was constant. PCB biotransformation rates by LB400 were obtained from sediment-free experiments.^33−35^ In the present study, PCB levels decreased in the presence of LB400 and saponin but were relatively unchanged in either vapor or aqueous phases in controls (i.e., without LB400). Thus, saponin only affected biotic processes and not abiotic. In previously conducted experiments including sediment, it was possible to observe somewhat higher biotransformation rates when saponin was present (unpublished data). Biotransformation rates from these experiments were only included in modeled results of bioaugmented treatments.

Four ordinary differential equations describe individual congener concentrations (PCB_i_) in aqueous and gas phases based on the PCB mass accumulated in SPME and PUF samplers, respectively, at each time point. Only five of the most abundant congeners were applied to the model (PCBs 4, 17, 19, 31, and 52). The equations were solved using the function code from the “R” package deSolve. The absorption–desorption rates were obtained from the non-bioaugmented controls and applied to modeled results of the bioaugmented treatments. Details of equations and parameters are shown in Section S5.

### Quality Assurance and Quality Control (QA/QC)

Extraction efficiency, reproducibility, and accuracy were assessed using surrogate standards, replicates of method blanks, and analysis of standard reference materials (SRMs). Method blanks were identical to each passive sampler but were not deployed in bioreactors. SRM analysis has been previously described.^33^ Briefly, the percent recovery of our measured values against the certified values for the 27 PCB congeners reportedly yielded a mean of 96 ± 8%. For the present study, mean and standard deviation percentage recoveries of PCB 14, PCB 65-d5, and PCB 166 in experimental samples (PUF) were 93 ± 15, 86 ± 11, and 91 ± 11%, respectively. Percentage recoveries of surrogate standards less than 100% were used to correct the congener mass as follows: PCB 14 recovery was used to correct PCB 1 to PCB 39, PCB 65-d5 was used to correct PCB 40 to PCB 127, and PCB 166 was used to correct PCB 128 to PCB 209 (sorted by IUPAC number). Samples were processed in batches of five along with one method blank per batch. All materials used in sample extraction had either been triple rinsed with solvent or combusted overnight at 450 °C to prevent background PCB contamination.

PCB mass detected in method blanks was used to determine the limit of quantification (LOQ) as the upper limit of the 95% confidence interval. The concentration dataset was dichotomized at the congener-specific LOQ: concentrations of congeners below their respective LOQ were treated as the LOQ divided by the square root of 2 (PCB_i_ = [LOQ_PCBi_]/√2).^68^

### Statistical Analysis

Statistical analyses were performed to determine which fixed effects (time, presence of LB400, and presence of saponin) significantly impacted LC-PCB accumulation in passive samplers. A three-way mixed-effects analysis was conducted by fitting fixed effects to a restricted maximum likelihood (REML) linear mixed-effects model in GraphPad Prism. The three-way test was followed up by a two-way mixed-effects analysis using consolidated data which combined groups with those that had statistically insignificant main effects (*p* > 0.05). Results of both mixed-effects analyses were followed up by multiple pairwise comparisons of means evaluated by Holm–Šídák-adjusted posthoc *t*-tests. The sum of LC-PCB congeners (∑[mono-, di-, and trichlorobiphenyls]) detected on passive samplers was natural log transformed before conducting statistical tests. Full results of statistical analyses are shown in Section S6.

## Results and Discussion

### PCB Emissions from Sediment to Air in Controls

We hypothesized that by simultaneously measuring PCB accumulation in vapor and aqueous phases within bioreactors using PUF and SPME passive samplers, respectively, we could observe temporal mass transport dynamics of labile PCBs. Congener profiles detected by each type of passive sampler in controls confirmed this hypothesis (Figure 1). PCBs collected on passive samplers reflect the labile congener distribution present in Altavista sediment (Figure 1A);^33^ PCBs collected on PUF passive samplers reflect a subset of the labile PCB fraction that desorbed from sediment particles and volatilized (Figure 1B). SPME fiber passive samplers immersed in sediment slurry captured the labile PCBs that desorbed from sediment particles but had not yet volatilized at the time of sampling (Figure 1C). The small difference between the congener distributions in the two passive sampling media are due to differential congener solid–water distribution coefficients and Henry’s constants.

In this study, our kinetic phase passive sampling approach allowed us to observe temporal mass transport dynamics for the inventory of congeners present in Altavista sediment. The Altavista sediment PCB profile was determined previously via whole sediment extraction and is enriched in LC-PCBs relative to the suspected source contamination (Aroclor 1248; Figure 1A).^33,55^ The presence of these LC-PCBs is evidence that anaerobic reductive dechlorination of sediment PCBs by native organohalide-respiring bacteria (OHRB) has already occurred.^55,69^ PCB congeners suspected to be reductive dechlorination products include PCB 1 (2-monochlorobiphenyl), PCB 4 (2,2′-dichlorobiphenyl), PCB 10 (2,6-dichlorobiphenyl), and PCB 19 (2,2′,6-trichlorobiphenyl; Figure 1A). These same PCB congeners are also some of the most volatile in the congener profile (Figure 1B).

### Mitigation of PCB Emissions from Sediment to Air Using*P. xenovorans* LB400

**Figure 2:**
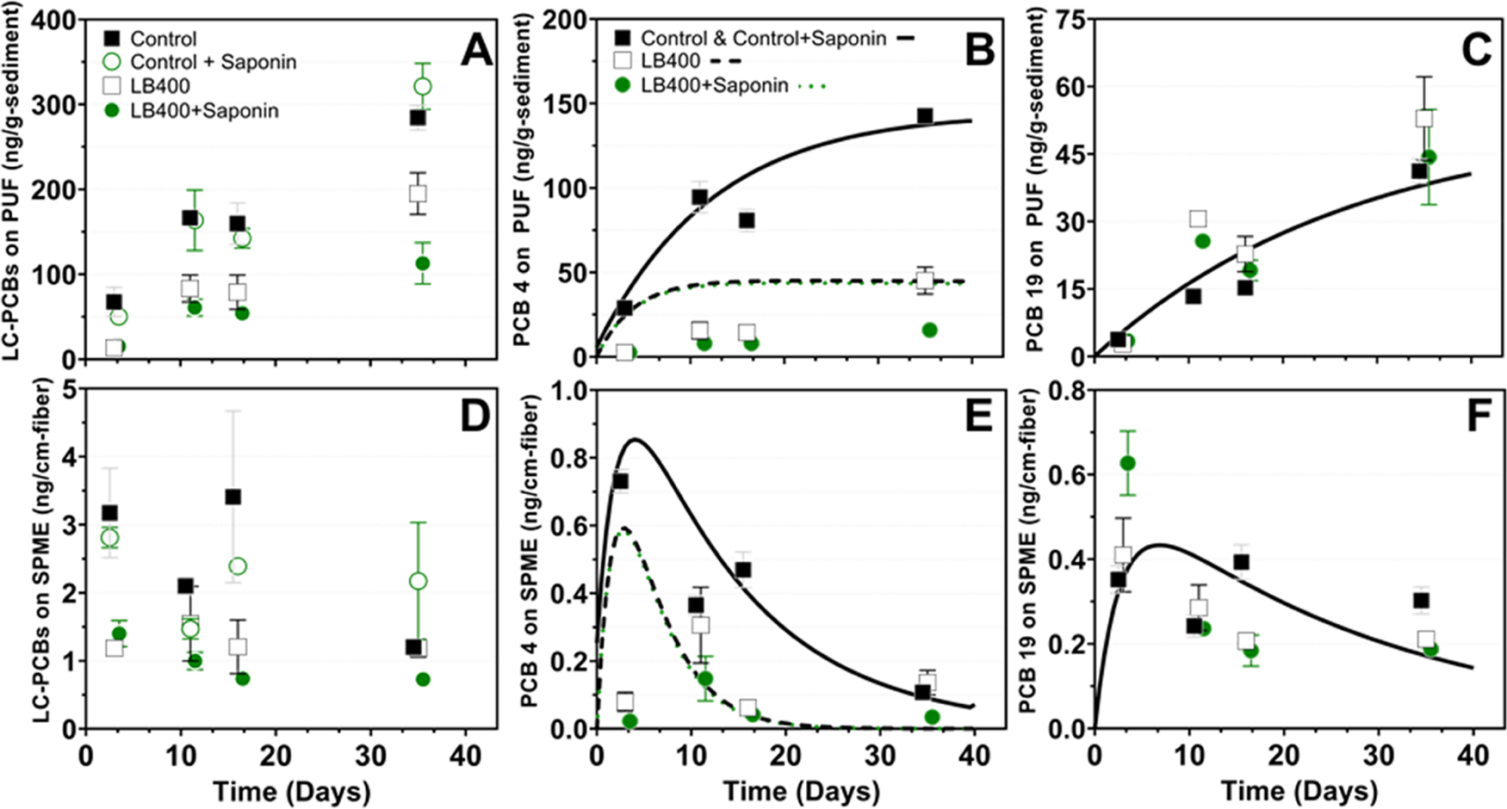
PCBs accumulated in vapor and free aqueous phases (top and bottom rows, respectively). Symbols indicate observed experimental results, and corresponding lines represent results of the reactive transport model. (A) LC-PCBs in vapor phase in all treatments. No significant differences were detected in controls with and without saponin, so those data were consolidated in individual congener plots. (B) PCB 4 (2,2′-dichlorobiphenyl) in vapor phase. On average, PCB 4 accumulation was reduced by 77% in LB400 treatments and 92% in LB400 + Sap treatments. (C) PCB 19 (2,2′,6-trichlorobiphenyl) in vapor phase. PCB 19 serves as an example of a double-*ortho*-substituted reductive dechlorination byproduct whose accumulation in PUF was not significantly reduced in bioaugmented treatments, relative to controls. (D) LC-PCBs in free aqueous phase. Measurements indicate that the freely dissolved mass of LC-PCBs was initially decreased in bioaugmented treatments compared to controls but remained constant throughout the incubation period. (E) PCB 4 in free aqueous phase. A 7-fold decrease in the initial amount freely dissolved PCB 4 mass at *T* = 3 days translated to a decrease in the amount accumulated in the vapor phase. (F) PCB 19 in free aqueous phase. The observed 54% decrease in freely dissolved mass across all treatments and controls can be attributed to volatilization because there was no significant difference at *T* = 3 days, as with PCB 4. The error bars represent the standard error of triplicate measurements in non-bioaugmented controls (*n* = 3) and quadruplet in bioaugmented treatments (*n* = 4).

**Figure 3:**
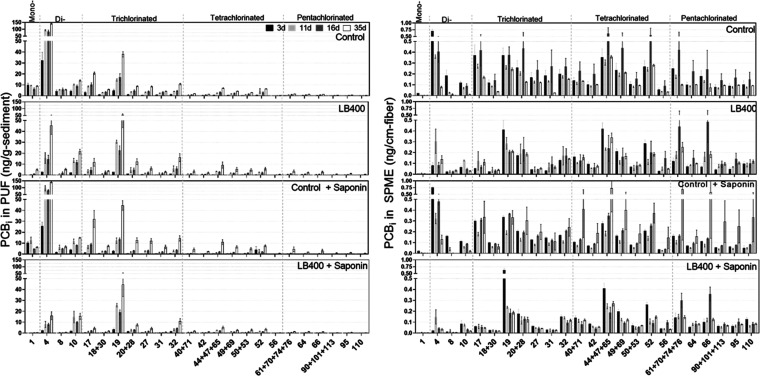
PCB_i_ accumulation in the vapor phase (left) and free aqueous phase (right) over the 35-day incubation period. Vapor phase profiles reveal that LB400 prevented 59% of the PCBs shown here from volatilizing. A small number of congeners were particularly resistant to biodegradation (e.g., PCBs 10,19, 32). Aqueous phase PCB profiles show a proportional decrease in freely dissolved concentrations for those congeners that volatilized. Taken together, these results indicate that LB400 biotransformed freely dissolved LC-PCBs as they desorbed from sediment particles and prevented them from volatilizing. The *T* = 16 days SPME measurement in the control is likely elevated due to small-scale sediment heterogeneity. The error bars represent the standard error of triplicate measurements in non-bioaugmented controls (*n* = 3) and quadruplet in bioaugmented treatments (*n* = 4).

The central hypothesis of this work was that LB400 could mitigate release of volatile PCBs from contaminated sediment to air. Overall, vapor measurements confirmed this hypothesis by showing that total PCB mass in bioaugmented treatments decreased by an average of 57% relative to non-bioaugmented controls, across all timepoints. Our comparative congener-specific analysis showed that LB400 was most effective at biodegrading LC-PCBs that also readily volatilized from sediment slurry in controls (Figures 2A and 3 and Table S4). We did not measure aerobic PCB degradation products (e.g., chlorobenzoates) or PCB uptake in biomass during these experiments to further document biodegradation processes but instead used comparisons between non-bioaugmented controls and bioaugmented treatments to account for the major alternative abiotic PCB loss mechanisms in the headspace and aqueous compartments of the bioreactor (i.e., volatilization and sorption, respectively). Thus, the observed differences between treatments and controls directly implicate biodegradation as the principal PCB loss mechanism in bioreactors containing biphenyl-grown LB400.

Comparison of LB400 bioaugmented treatments with non-bioaugmented controls shows how specific LC-PCBs are biodegraded (e.g., PCB 4), whereas other LC-PCBs are not (e.g., PCB 19). In the LB400 treatments, the amount PCB 4 in the vapor phase decreased by 77% compared to controls (*T* = 35 days), while in the LB400 + Sap treatment the amount PCB 4 in the vapor phase decreased by 92% at the same timepoint (Figure 2B). In contrast, volatilization of PCB 19, the second-most abundant congener, was not affected by bioaugmentation, compared to controls (Figure 2C). Although LB400 has broad congener specificity because it can attack PCBs at both 3,4- and 2,3-positions,^70^ LB400 cannot degrade certain double-*ortho*-substituted congeners (e.g., PCB 19) and is weaker against double-*para-*substituted congeners (e.g., PCB 28; 2,4,4′-trichlorobiphenyl).^27,71−73^

PCB 4 is the most abundant congener in the sediment profile and in the control bioreactor headspace (∼10 and ≥45%, respectively; Figure 1). Its disproportionate contribution to profiles detected in the vapor and free aqueous phases indicates that PCB 4 is more mobile in the environment than its HC-PCB parent congener(s) and thus more likely to participate in the PCB inhalation exposure pathway.^19^ PCB 4′s rapid volatilization from contaminated sediments in the absence of LB400 is troubling because it is a potent neurotoxicant among other di-*ortho*-substituted congeners that have been assayed.^74,75^ The congener’s outsized contribution to the gas-phase profile may be attributed to its non-coplanar molecular structure, which prevents it from binding with the organic matter in sediment as easily as coplanar congeners belonging to the same homolog group.^76,77^

**Figure 4:**
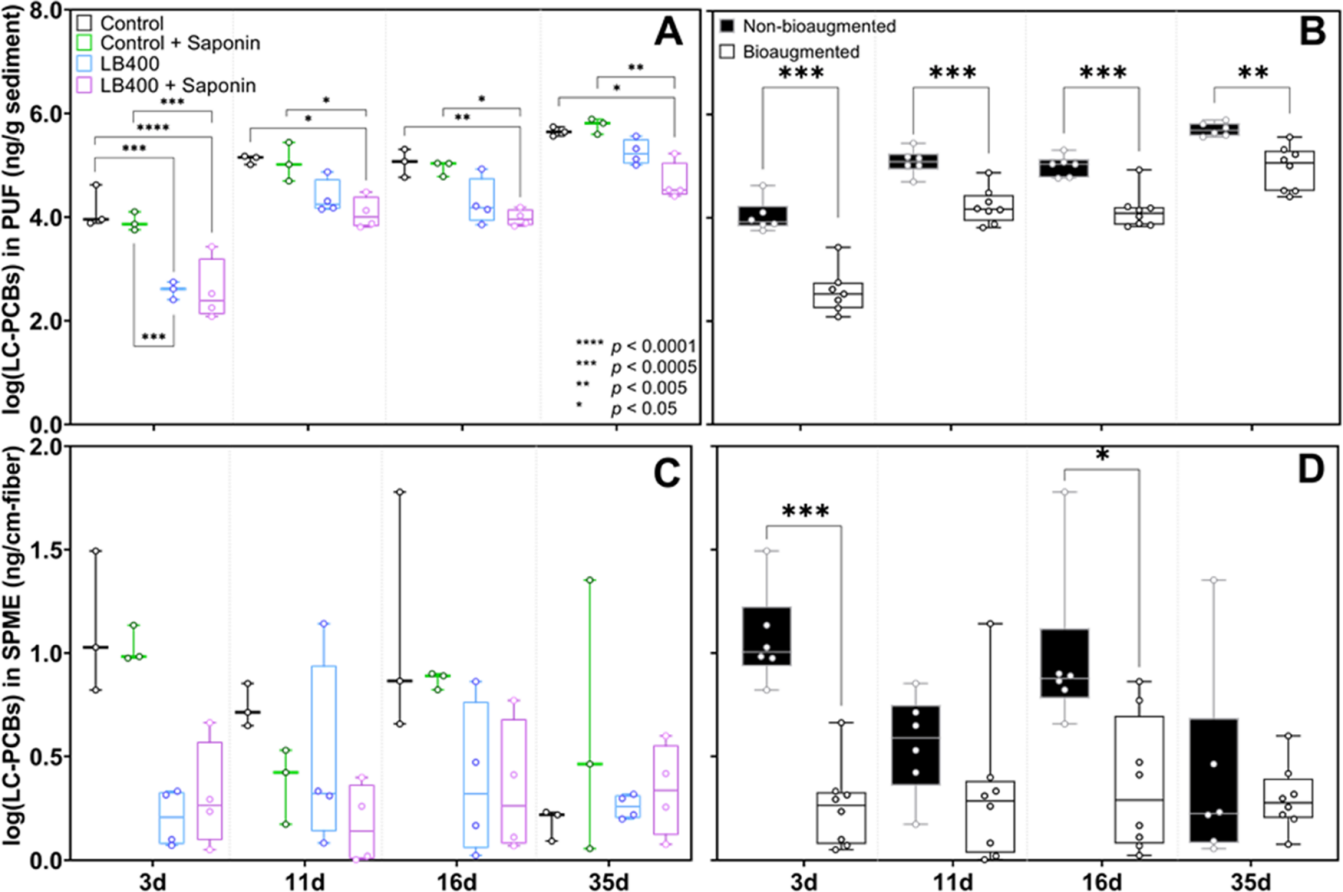
(A) Results of three-way mixed-effects statistical analysis on log-transformed measurements of vapor phase LC-PCBs using LB400, saponin, and time as fixed effects fitted to a restricted maximum likelihood (REML) linear mixed-effects model. (B) Results of a two-way mixed-effects analysis of the vapor-phase PCBs. Non-bioaugmented controls and bioaugmented treatments were consolidated into two groups because saponin was an insignificant main effect. Bioaugmented treatments are highly significantly different than non-bioaugmented. (C) Results of three-way statistical analysis of free dissolved PCB concentrations. No significant differences in aqueous LC-PCBs were detected between bioaugmented treatments and non-bioaugmented controls. (D) Results of two-way statistical analysis using consolidated aqueous phase data. Once data was consolidated, a highly significant difference (*p* < 0.0005) between bioaugmented treatments and controls was detected at *T* = 3 days. All mixed-effects analysis results were evaluated by multiple pairwise comparisons using Holm–Šídák-adjusted posthoc *t*-tests. Only significant differences are indicated although comparisons were made between each treatment at every timepoint. The whiskers range from the lowest to the highest observed values (i.e., “min-to-max”).

Aqueous phase measurements displayed an initial “flush” of labile PCBs from sediment, which was significantly decreased when LB400 was present (Figures 2D, 3, and 4C, 4D, and Table S5). This phenomenon is exemplified by a 7-fold decrease in the freely dissolved mass of PCB 4 in the bioaugmented treatment at *T* = 3 days, compared to the controls (Figure 2E). Aqueous measurements also showed that the freely dissolved mass of certain PCBs declined more slowly, relative to PCB 4, even when LB400 was present (Figure 3). This slower decrease in the aqueous phase was attributed to volatilization for double-*ortho-*substituted congeners that have been documented as resistant to LB400-mediated biodegradation (e.g., PCB 10 and 19; Figure 2F).

Consistent with our previous work,^33^ limitations in LB400′s PCB congener specificity are further distinguishable by observation of individual PCBs that were not removed from the aqueous phase and/or prevented from volatilizing from sediment slurry to the vapor phase (Figure 3 and Table S4). For example, PCB 17 (2,2′,4) is a congener highly amenable to biodegradation by LB400 whereas PCB 10 (2,6′) is not. The same dynamic is demonstrated by PCB 31 (2,4′,5) vs PCB 32 (2,4′,6; Figure 3). The observation that certain PCBs (e.g., PCB 10, PCB 19, PCB 32) were not removed in the presence of LB400 also suggests that sorption of PCB congeners to LB400 cells was not a significant PCB loss mechanism in these experiments. The persistence of the PCBs 20 + 28 (2,3,3′ + 2,4,4′) coelution in the aqueous phase over the incubation period further illustrates LB400′s difficulty in degrading double-*para-*substituted congeners (Figure 3). Occurrence and persistence of PCB 28 is noteworthy because it has been detected at relatively high concentrations in water, air, and vegetation surrounding the New Bedford Harbor Superfund site.^5,18,78^ Further, it makes up nearly half the relative congener abundance in the exposure profile detected in human serum from the MARBLES cohort—a prospective study of pregnant women in northern California at increased risk for having a child with a neurodevelopmental disorder.^79^ Resistance of certain LC-PCBs (PCBs 10, 19, 28) and their metabolites to biodegradation has important implications for site-specific risk assessment and remedial design.

Our findings suggest that displacing sediment in highly PCB-contaminated waterways (i.e., dredging) without a strategy to degrade or capture PCBs that become freely dissolved and then volatilize may increase inhalation exposure risk to surrounding communities and workers. Results show how double-*ortho*-substituted dechlorination byproducts, which are broadly resistant to aerobic biodegradation (e.g., PCBs 10 and 19) may be emitted at considerably higher levels than other congeners with similar relative abundance in sediment following mechanical perturbation, as well as those specifically resistant to LB400-mediated biodegradation (PCB 28; Figures 1 and 3). For example, PCB 19 is equally abundant to PCB 31 in Altavista sediment (∼4.8% by mass; Figure 1A) but has a relative contribution to the air profile that is ∼6 times higher (12.5% vs 2%, respectively; Figure 1B). Conversely, our observations also suggest that LB400 could be used for targeted bioremediation of well-characterized contaminated sites where reductive dechlorination in sediments is ongoing or known to have occurred and produced LC-PCBs more amenable to biodegradation, such as PCBs 1, 4, 8, and 17 (Figure 3 and Table S4). This finding indicates that, in some cases, LB400 is a viable bioaugmentation candidate to use either as an alternative to or in combination with dredging to reduce emissions of certain LC-PCBs during and immediately after perturbation of contaminated sediment. However, our bioreactor experiment does not demonstrate whether LB400 bioaugmented in liquid culture alone can completely block sediment–air PCB emissions over large areas and longer timescales. This offers insight to one remediation approach at sites where atmospheric release of PCBs from sediment is, or may become, a concern.

### Effect of Saponin on PCB Bioavailability and Biodegradation by LB400

We hypothesized that saponin would increase PCB bioavailability via micelle-facilitated desorption from sediment particles. Micelles are spherical, supramolecular assemblies which form when the biosurfactant monomers reach their critical micelle concentration (CMC) in solution. Micelle-facilitated desorption occurs when organic compounds, such as PCBs, are solubilized through assimilation into the hydrophobic core of the micelles. We tested our hypothesis that saponin would increase desorption of LC-PCBs from sediment by comparison of vapor and aqueous phase PCBs in non-bioaugmented flasks (i.e., Control + Sap vs Control; Figure 4). Similarly, we hypothesized that adding saponin to bioreactors concomitantly with LB400 would facilitate decreases in vapor and aqueous phase PCBs, compared to bioreactors with LB400 alone (i.e., LB400 + Sap vs LB400; Figure 4). These comparisons were statistically evaluated by a three-way mixed-effects analysis using time, presence of saponin, and presence of LB400 as fixed effects (Figure 4A, 4C). Interaction of fixed effects was also evaluated. Our findings suggest that saponin did not significantly affect PCB desorption (i.e., bioavailability) and/or their subsequent volatilization because neither vapor nor aqueous measurements in Control + Sap were significantly different than Control at any timepoint (*p* > 0.05; Figure 4A, 4C and Tables S9 and S15). Additionally, the three-way statistical analysis indicated that the presence of saponin was a statistically insignificant main effect (*p* > 0.05) on the amount of LC-PCBs in both the vapor and aqueous phases (Tables S7 and S13). Detailed information about the mixed- and fixed-effects statistical analyses is in Section S6.

In contrast, pairwise comparisons of vapor phase PCBs at each timepoint revealed that LB400 + Sap was significantly different (*p* < 0.05) than Control and Control + Sap at all timepoints. LB400 alone was significantly different from the Control only at *T* = 3 days (Figure 4A). Moreover, congener-specific analysis of vapor phase PCBs showed that, on average, LB400 + Sap was 24% more effective at preventing volatilization of many of the most abundant congeners throughout the incubation period than LB400 alone (Table S4). These observations suggest that saponin did have some beneficial effect on biodegradation of PCB congeners by LB400; however, interaction of LB400 and saponin was statistically insignificant according to the three-way mixed-effects analysis (*p* = 0.1014 and 0.941 for the vapor and aqueous phases, respectively; Tables S7 and S13).

We performed a two-way follow-up analysis by consolidating control and treatment data from four groups into two (bioaugmented and non-bioaugmented) after saponin was found to be an insignificant main effect. Organizing the data in this way allowed us to conduct the same statistical tests using only time and presence of LB400 as fixed effects. The follow-up analysis revealed highly significant differences (*p* < 0.0005; Table S11) between combined bioaugmented treatments (LB400 and LB400 + Sap) and combined non-bioaugmented controls (Control and Control + Sap) at 3–4 timepoints in the vapor phase and at *T* = 3 days in the aqueous phase (Figure 4B, 4D). This outcome reaffirms the principal conclusion of this work: bioaugmentation with LB400 decreases volatilization of LC-PCBs from sediment slurry via biodegradation as they desorb from sediment particles and become freely dissolved.

**Figure 5:**
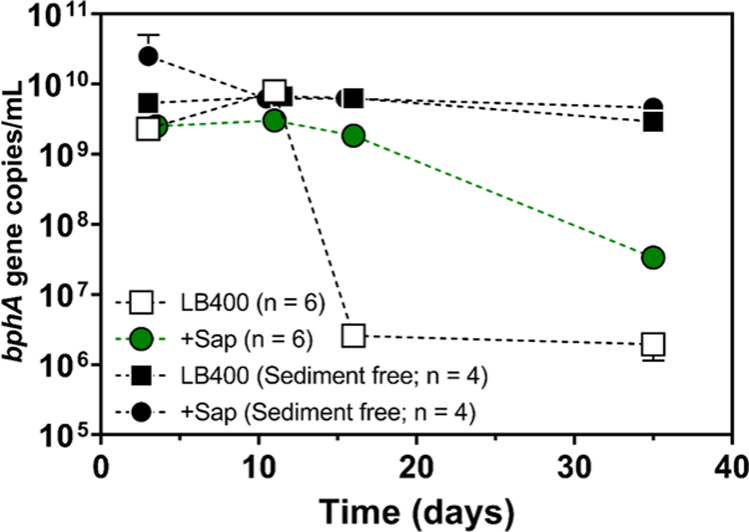
Abundance of biphenyl dioxygenase (*bphA*) genes in bioaugmented reactors over the 35-day incubation period (gene copies/mL slurry). The *bphA* abundance in the saponin-free treatment dropped 3 orders of magnitude after 11 days, whereas the saponin-amended treatment remained level until 16 days. The error bars represent standard error of six replicates (*n* = 6). Symbols obscure the error bars where they are not visible.

The effect of sediment, PCBs, and saponin on biphenyl dioxygenase gene (*bphA*) abundance in bioaugmented bioreactors was examined with qPCR. The *bphA* abundance in sediment-free LB400 controls was remarkably stable over the 35-day experiment compared to bioreactors containing sediment (Figure 5). Elevated *bphA* levels were seen at *T* = 3 days in sediment-free + Sap controls suggesting that saponin might be used as a growth substrate for LB400. An approximately 4 orders of magnitude drop in *bphA* abundance in LB400 reactors with sediment suggests that the sediment (and/or PCBs) is detrimental to LB400 cells. Interestingly, the *bphA* abundance in LB400 + Sap (with sediment) was 3 orders of magnitude greater than LB400 alone at *T* = 16 days (Figure 5). This suggests that saponin somehow protected LB400 cells in the presence of sediment and PCBs. Differences in *bphA* abundance between the LB400 and LB400 + Sap treatments might be explained by LB400 using saponin for growth although that effect might have only lasted a few days according to the sediment-free controls.

PCB- (and PAH-) degrading microorganisms have been reported to use saponin as a growth substrate, although, at concentrations much higher than those used in this study (1–15 g/L).^41,80^ We tested if LB400 could grow on 500 mg/L saponin as its sole carbon and energy source. This test was meant to determine whether saponin’s effect on PCB levels could be reasonably attributed to its role as a growth substrate for LB400 to maintain cell numbers in sediment. Results showed an initial LB400 cell density increase after 1 day followed by a gradual decrease over the next 6 days (Figure S4); this growth pattern supports the idea that LB400 can grow on saponin and also aligns with qPCR results. Alternatively, saponin is known to alter cell surface hydrophobicity (CSH) and surface charge (ζ-potential) which both influence microorganism–substrate interactions.^81,82^ However, the overall effect of these changes on biodegradation are highly strain and substrate specific.^81−84^ Based on elevated LB400 cell density when incubated with saponin (Figure S4) and elevated *bphA* abundance in sediment-free reactors (Figure 5), elevated LB400 cell numbers as a result of growth on saponin is a possible explanation for improved biodegradation performance in the LB400 + Sap treatment (Figure 5). Although changes in cell surface properties may have also occurred in this experiment, further research outside the scope of this study is required to determine the mechanism of saponin’s protective effect on LB400 in PCB-contaminated sediment slurry.

The abundance of *bphA* in non-bioaugmented sediment controls was not measured in this study. Previous qPCR analysis of Altavista lagoon sediment samples showed an average *bphA* abundance of 9.4 × 10^5^ copies/g sediment.^55,57^ The possible contribution of naturally occurring aerobic PCB-degrading bacteria to PCB biodegradation in the treatments is likely overshadowed by the >5 orders of magnitude population of active (biphenyl-grown) LB400 cells after bioaugmentation. The experimental design allows comparisons of non-bioaugmented sediment with bioaugmented sediment so that any potential contributions of native PCB-degrading bacteria are accounted for. Previous characterization of the Altavista sediment microbial community structure revealed a diverse microbial community dominated by Proteobacteria, Firmicutes, and Chloroflexi phyla.^55,56^ Potential interactions of LB400 with other microbial community members could influence bioaugmentation success and should be considered in future work.

Coupling bioaugmentation with other remediation techniques, such as black carbon sequestration, may be necessary to deliver active populations of PCB-degrading microorganisms to contaminated aquatic environments. In other studies, LB400 was an efficient PCB-degrader when incubated in coculture with black carbon and anaerobic OHRB in mesocosm studies and in pilot-scale applications using bioamended activated carbon as a sediment delivery vehicle.^42−44^ This is an appealing approach because cells introduced to an overlying water column in liquid culture alone would likely become too dilute at the field scale for effective biodegradation to occur. Furthermore, our data suggest that biphenyl-grown LB400 is most effective at mitigating PCB emissions when the cells are most active (i.e., at the beginning of the experiment) and become less effective over time in Altavista sediment (Figures 2A, 3, and 5). Thus, delivering and sustaining PCB degraders as biofilms on the surfaces of black carbon materials, such as activated carbon, may be the most viable noninvasive sediment PCB bioremediation strategy presently available. By using this combination of techniques, our approach may be further developed to help meet regulatory PCB-cleanup standards and contain releases of even the most recalcitrant LC-PCB congeners via sequestration in the absence of a microorganism or consortia with a “one-size-fits-all” enzyme complex.

### Results of PCB Reactive Transport Model

Measurements taken in our experiment were consistent with results from a reactive transport model developed and optimized using bioreactor parameters, experimental conditions, and sediment characteristics specific to this study.^67^ The model simulated congeners representative of overall system dynamics and compared results to experimental observations (Figure 2). Simulated results for these congeners are shown in Figure S5. Parameters which most greatly influenced modeled results were related to absorption–desorption kinetics of PCBs into the aqueous phase from suspended sediment particles. Both experimental and modeled results showed that LB400 significantly decreased the initial amount of freely dissolved PCBs at *T* = 3 days, which resulted in a significantly lower amount of volatilized mass.

In the model, we used biotransformation rates obtained from previously conducted sediment-free experiments which caused simulated outputs to match well with experimental results for the LB400 treatments, especially for PCBs in the vapor phase.^33^ However, the only biotransformation rates available for LB400 + Sap were obtained from experiments with sediment (unpublished data), but these did not match well with values observed in the present study. We used data from previously conducted experiments to calculate biotransformation rates to be used in the model because it was impossible to do so with the passive sampling methods we employed in this experiment. All other input constants and coefficients were obtained from literature values for conditions similar to this study or were fitted using the “R” script we developed in conjunction with data from controls in this study (Table S6). From model results, it seems that the LB400 + Sap biotransformation rate should have been at least 3–5 times greater than what we calculated based on previous unpublished experiments. However, modeled results aligned well with experimental observations. The greatest differences between observed and modeled results occurred at the beginning and end of the experiment when LB400 cells were most and least active, respectively. Application of this model to our lab-scale study demonstrates the utility of PUF and SPME passive samplers to monitor airborne PCB emissions by simultaneously measuring congeners in air and water during pilot and field-scale remediation activities. These measurements, in combination with reactive transport models like the one developed here, are the first steps in estimating potential community exposure in inhalation risk assessments from semi-volatile PCBs during ex situ remediation activities, like dredging.

## Supporting Information Available

Additional information about sediment and site description; experimental design and passive sampling; qPCR parameters; experimental results; reactive transport modeling; statistical analysis; and PCB quantification (PDF)

## Notes

Underlying data for this work^66^ and the “R” code for the PCB reactive transport model^67^ have been deposited in the Iowa Research Online (IRO) institutional data repository for future reuse under an Open Data Commons Attribution License (ODC-By) and an Open Software License (OSL 3.0), respectively. There is no user registration or fee requirements to download the underlying dataset or mass transport model developed for this study.
