# Supplementary material for: Aerobic Bioaugmentation to Decrease Polychlorinated Biphenyl (PCB) Emissions from Contaminated Sediments to Air
Source: Environ Sci Technol. 2022 Sep 30;56(20):14338–49. doi: 10.1021/acs.est.2c01043 (PMC9583607; doi:10.1021/acs.est.2c01043)
Supplement: Supplementary file 1 — es2c01043_si_001.pdf [file es2c01043_si_001.pdf]

Supporting Information

Aerobic Bioaugmentation to Decrease Polychlorinated Biphenyl (PCB) Emissions from Contaminated Sediments to Air

*Christian M. Bako, Andres Martinez, Jessica M. Ewald, Jason B.X. Hua, David J. Ramotowski, Qin Dong, Jerald L. Schnoor, and Timothy E. Mattes**

The Department of Civil & Environmental Engineering, 4105 Seamans Center for the Engineering Arts & Sciences, University of Iowa, Iowa City, IA United States, 52245

IIHR-Hydroscience & Engineering, University of Iowa, Iowa City, IA 52242

*Corresponding author; Email – tim-mattes@uiowa.edu; Phone – (319) 335-5065

21 pages: 6 Figures and 19 Tables

Contents:

Supporting Information....1
S1. Sediment & Site Description....3
S2. Experimental Design and Passive Sampling....4
S3. *bphA* Gene Abundance with qPCR....6
S4. *Paraburkholderia xenovorans* LB400 Growth on Saponin....6
S5. Mitigation of PCB Emissions from Sediment to Air Using *Paraburkholderia xenovorans* LB400....7
S6. PCB Reactive Transport Modeling....9
S6.1. Mass-Balance Equations for Reactive Transport Model....9
S6.2. Passive Sampler Mass-Balance Equations for Reactive Transport Model....9
S6.3. Definitions of Terms Used in Reactive Transport Model Equations....10
S6.4. Results of PCB Reactive Transport Model for Most Abundant PCB Congeners....12
S7. Statistical Analyses....13
S7.1. Summary of Results for LC-PCB Statistical Analyses....13
S7.2. Tabular Results for Statistical Analysis on PUF Measurements....15
S7.3. Tabular Results for Statistical Analysis on SPME Measurements....17
S8. PCB Quantification....19
References....20

Figures

Figure S1- Plan view map of the emergency overflow lagoon at the Altavista, VA wastewater treatment plant showing approximate PCB concentrations at previous sampling sites. 4
Figure S2 - Experimental design matrix for this study. 5
Figure S3 – Bioreactor utilized in this study design.. 5
Figure S4 – Accumulation of the most abundant individual congeners on passive samplers deployed in bioreactors. 12
Figure S5 – Residual, homoscedasticity, and QQ plots for results of three-way mixed-effect analyses of PUF samples (left) and SPME samples (right). 14

Tables

Table S1- Results of sediment characterization tests S3
Table S2 – Oligonucleotide primer information for the bphA target gene S6
Table S3 – Pertinent qPCR parameters in accordance with MIQE guidelines S6
Table S4 – % difference of PCB_i sediment-air emissions between treatments in PUF samples (ΔPCB_i)..S8
Table S5 – % difference of freely dissolved PCB_i between treatments in SPME samples (ΔPCB_i). S9
Table S6 – Definitions of each term used in the mass-balance equations for the reactive transport model and their dependent equations. S11
Table S7 – Tabular results of three-way mixed effects analysis on log-transformed LC-PCB PUF data S17
Table S8 – Tabular results of two-way mixed effects analysis on consolidated LC-PCB PUF data. S17
Table S9 – Results of Holm-Šídák's multiple comparisons tests following three-way mixed effects analysis on log-transformed LC-PCB PUF measurements. S17
Table S10 – Details of Holm-Šídák's multiple comparisons tests following three-way mixed effects analysis on log-transformed LC-PCB PUF measurements. S18
Table S11 – Results of Holm-Šídák's multiple comparisons tests following two-way mixed effects analysis on consolidated LC-PCB PUF measurements. S18
Table S12 – Details of Holm-Šídák's multiple comparisons tests following two-way mixed effects analysis on consolidated LC-PCB PUF measurements. S18
Table S13 – Tabular results of three-way mixed effects analysis on log-transformed LC-PCB SPME data. S19
Table S14 – Tabular results of two-way mixed effects analysis on consolidated LC-PCB SPME data ..S19
Table S15 – Results of Holm-Šídák's multiple comparisons tests following three-way mixed effects analysis on log-transformed LC-PCB SPME measurements. S19
Table S16 – Details of Holm-Šídák's multiple comparisons tests following three-way mixed effects analysis on log-transformed LC-PCB SPME measurements. S20
Table S17 – Results of Holm-Šídák's multiple comparisons tests following two-way mixed effects analysis on consolidated LC-PCB SPME measurements S20
Table S18 – Details of Holm-Šídák's multiple comparisons tests following two-way mixed effects analysis on consolidated SPME measurements S20
Table S19 – PCB precursor and product masses of labeled and unlabeled calibration standards employed in multiple reaction monitoring mode on the triple quadrupole mass spectrometer.[a] S21

S1. Sediment & Site Description

Table S1- Results of sediment characterization tests conducted by Minnesota Valley Testing Laboratory (MVTL, New Ulm, MN).

Soil Parameters	
Organic Carbon (%)	3.0
Nitrogen (N-NO_3)	12
Phosphorus (P) Meh 3 ppm	189
Potassium (K) Meh 3 ppm	52
Zinc (Zn) ppm	7.9
Sulfur (SO_4-S) ppm	95.3
Calcium (Ca) ppm	884.2
Magnesium (Mg) ppm	120.8
Boron (B) ppm	0.64 L
Iron (Fe) ppm	87.5 S
Manganese (Mn) ppm	40.0 S
Copper (Cu) ppm	7.6 S
Sodium (Na) ppm	32
pH	6.4
Buffer Index	7.1
CEC ($cmol_c$/kg)	6.0
Base Saturation (%):	
Calcium (Ca)	73.6
Magnesium (Mg)	16.8
Potassium (K)	2.3
Sodium (Na)	2.4
Hydrogen (H)	4.8
Texture (%):	Sandy Loam
Sand	72.2
Silt	24.7
Clay	3

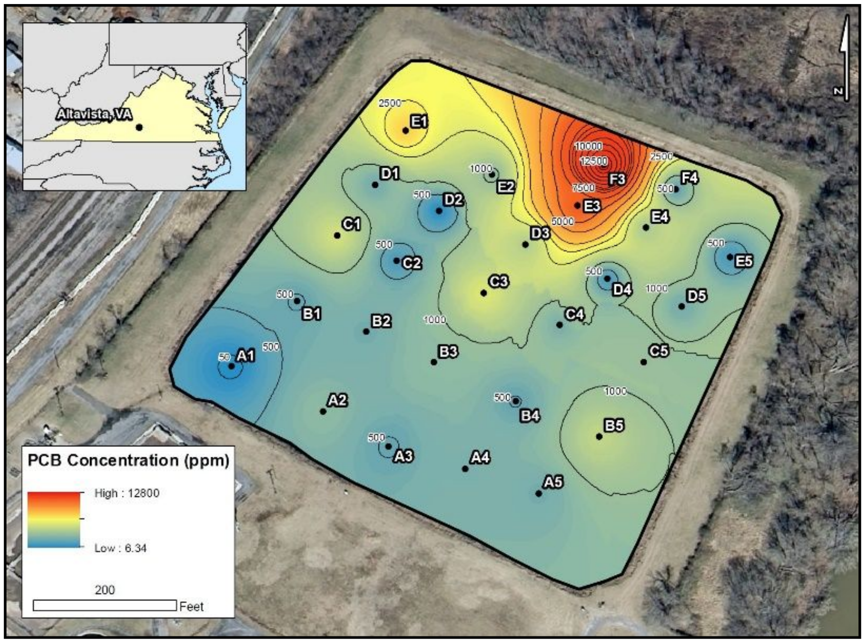

Figure S1- Plan view map of the emergency overflow lagoon at the Altavista, VA wastewater treatment plant showing approximate PCB concentrations at previous sampling sites.[1] PCB concentration contours were estimated using inverse distance weighted (IDW) interpolation. Map made using ArcGIS 10.4.1 (ESRI, Redlands, CA). Map courtesy of Reid Simmer (2018).

S2. Experimental Design and Passive Sampling

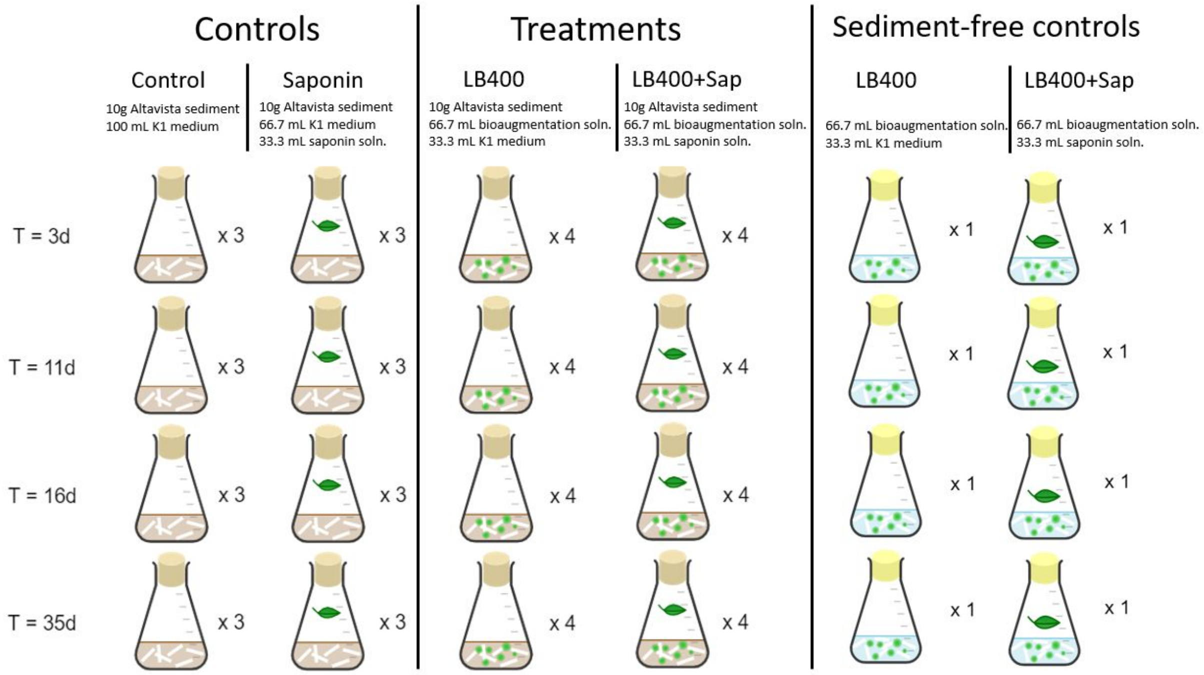

Figure S2 - Experimental design matrix for this study. Three replicates (n=3) used in control groups and four replicates (n=4) in bioaugmented treatments to account for possible variability in biological activity. Sediment-free controls with and without saponin were established for comparing levels of *bphA* gene abundance in treatments with qPCR.

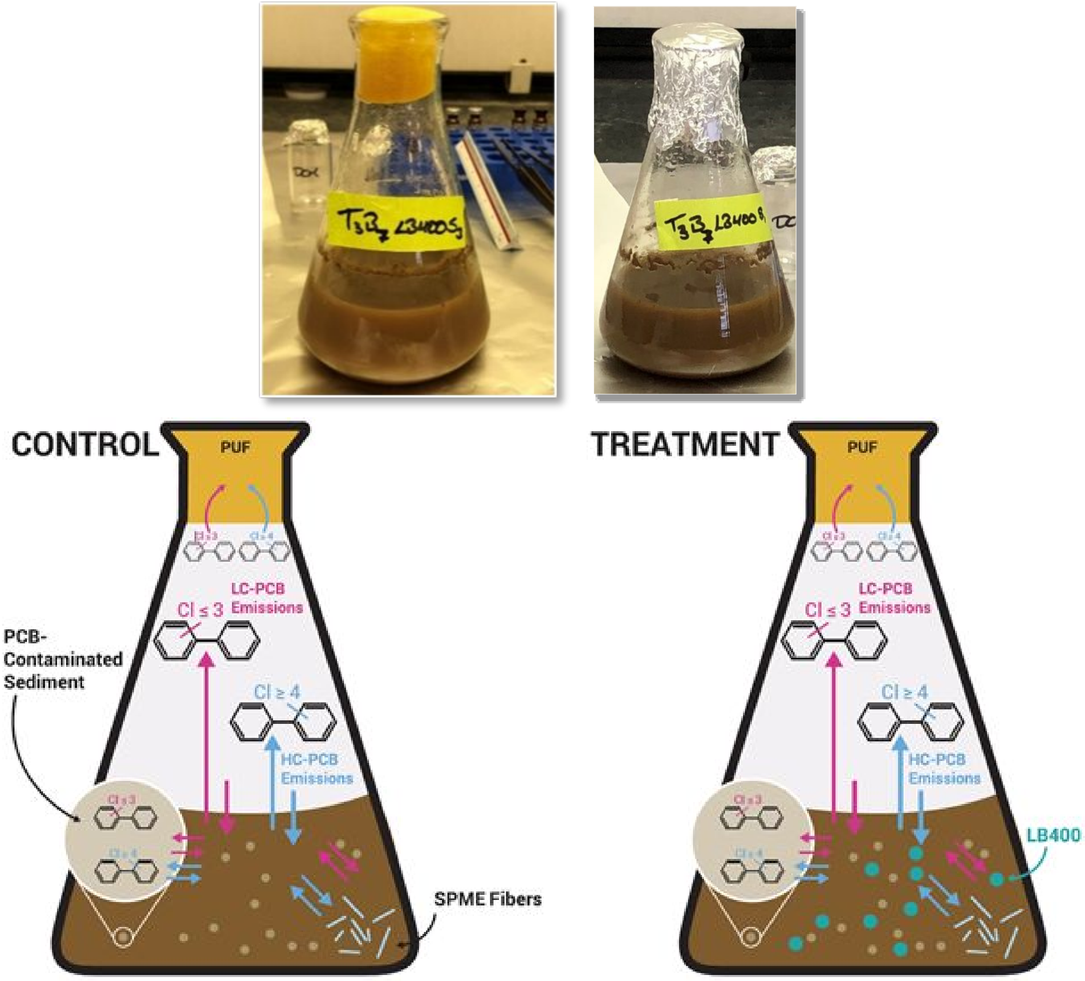

Figure S3 Bioreactor utilized in this study design. **Top left:** Shown with aluminum foil cover removed. **Top right:** Shown with aluminum foil cover. **Bottom:** Conceptual diagram of PCB mass transport dynamics within the bioreactor.

S3. *bphA* Gene Abundance with qPCR

The following tables contain primer and QA/QC details that satisfy MIQE guidelines for qPCR.

Table S2 – Oligonucleotide primer information for the *bphA* target gene.[2]

**Target gene**	**Primer name**	**Sequences (5'- 3')**	**Product size (bp)**	**Reference**
*bph*A (qPCR)	*bphA* 463f	CGCGTSGMVACCTACAARG	211	(Petrić et. al., 2011)
	bphA 674r	GGTACATGTCRCTGCAGAAYTGC		

Table S3 – Pertinent qPCR parameters in accordance with MIQE guidelines.[3]

Target gene	Primer concentration (µM)	DNA template mass (ng)	qPCR linear range (gene copies/reaction)	qPCR efficiency	Y-intercept
bphA	0.3	10	$30 - 30 \times 10^7$	97.58%	36.64

S4. *Paraburkholderia xenovorans* LB400 Growth on Saponin

An experiment was conducted to determine if *Paraburkholderia xenovorans* LB400 could use saponin (500 mg/L) as its sole carbon and energy source for growth. Biphenyl-grown LB400 was harvested and introduced into liquid K1 medium at an original $OD_{600} = 0.1$. Cells were washed prior to transfer to prevent residual biphenyl carryover. Growth was monitored by taking OD_{600} measurements and comparing to a saponin-free control (**Figure S4**).

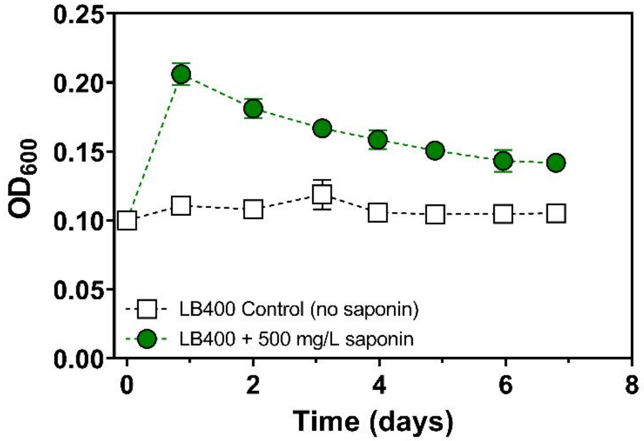

Figure S4 – Growth of LB400 with and without 500 mg/L saponin as the sole carbon and energy source in liquid K1 medium over 7 days. LB400 cells initially grew to $OD_{600} = 0.2$ after 1 day in the presence of saponin but dropped gradually to $OD_{600} = 0.15$ after 7 days. Error bars represent standard deviation of duplicate measurements.

S5. <u>Mitigation of PCB Emissions from Sediment to Air Using *Paraburkholderia xenovorans* LB400</u>

Table S4 – Percent difference of PCB_i sediment-air emissions between treatments in PUF samples (ΔPCB_i). Values shown are the percent differences between PCB_i's geometric mean in the treatments listed in the column heading (1-[GeomeanTreatment1/GeomeanTreament2]). Positive values (blue) indicate a lower amount of PCB_i mass accumulated in PUF of the first treatment, relative to the second, at each respective timepoint. Negative values (red) indicate higher PCB_i mass accumulated. LB400 mitigated nearly all release of mono- and dichlorinated congeners from sediment slurry to PUF. Presence of saponin enhanced emissions mitigation by approximately 24% throughout the incubation period, relative to the saponin-free treatment. Conditional color formatting uses maximum value of 100%, minimum of -100%, and midpoint of 0%.

PUF

	PCB_i	ΔPCB_i between LB400 and Control				ΔPCB_i between LB400+Sap. and Control				ΔPCB_i between LB400+Sap. and LB400				ΔPCB_i between Sap and Control			
		3d	11d	16d	35d	3d	11d	16d	35d	3d	11d	16d	35d	3d	11d	16d	35d
**Mono-**	1	99%	97%	84%	47%	98%	98%	92%	94%	-90%	33%	53%	89%	-5%	-28%	23%	30%
**Di-**	4	92%	86%	83%	70%	93%	93%	90%	90%	15%	52%	40%	65%	18%	1%	0%	2%
	8	95%	80%	78%	9%	95%	91%	91%	84%	11%	53%	62%	83%	83%	-1%	11%	-16%
	10	51%	-24%	-32%	-50%	59%	-19%	-14%	-6%	16%	4%	13%	29%	8%	-8%	8%	-7%
**Tri-**	17	74%	69%	65%	50%	74%	86%	78%	80%	1%	55%	37%	61%	52%	29%	15%	-47%
	18+30	69%	67%	50%	38%	70%	85%	72%	70%	2%	55%	44%	51%	31%	26%	21%	-25%
	19	37%	-112%	-33%	-34%	35%	-77%	-14%	-9%	-4%	17%	14%	18%	36%	18%	18%	-17%
	20+28	-15%	3%	6%	-30%	-27%	33%	33%	17%	-10%	31%	29%	36%	49%	50%	32%	-39%
	27	-124%	57%	44%	35%	-145%	66%	51%	54%	-9%	20%	14%	30%	65%	25%	18%	-35%
	31	55%	69%	70%	36%	54%	86%	83%	76%	-1%	56%	43%	62%	41%	43%	32%	-51%
	32	2%	-22%	-27%	-39%	-9%	32%	1%	4%	-10%	44%	22%	31%	43%	30%	18%	-31%
**Tetra-**	40+71	40%	-45%	-15%	-49%	25%	-23%	3%	12%	-25%	15%	16%	41%	34%	56%	31%	-78%
	42	48%	-33%	27%	-26%	41%	-6%	42%	24%	-14%	20%	20%	40%	24%	48%	42%	-49%
	44+47+65	43%	-36%	14%	-15%	35%	-9%	30%	31%	-14%	20%	19%	40%	41%	53%	30%	-51%
	49+69	56%	-6%	33%	-9%	53%	22%	50%	38%	-7%	26%	25%	44%	27%	53%	33%	-52%
	50+53	-17%	33%	-1%	-42%	-17%	47%	18%	10%	0%	22%	19%	37%	43%	45%	29%	-58%
	52	37%	31%	34%	16%	20%	52%	53%	49%	-26%	30%	28%	39%	-145%	44%	27%	-18%
	56	20%	-5%	8%	-57%	16%	40%	30%	18%	-6%	43%	24%	48%	40%	82%	41%	-77%
**Penta-**	61+70+74+76	33%	19%	16%	-71%	37%	44%	40%	18%	6%	30%	29%	52%	24%	72%	35%	-104%
	64	44%	7%	0%	-54%	37%	33%	25%	7%	-13%	28%	25%	39%	30%	52%	42%	-31%
	66	28%	-2%	15%	-76%	22%	28%	39%	10%	-8%	29%	28%	49%	62%	81%	44%	-82%
	90+101+113	58%	62%	25%	-34%	48%	64%	39%	29%	-23%	7%	19%	47%	-5%	50%	23%	-67%
	95	63%	41%	10%	-57%	51%	58%	30%	9%	-33%	28%	22%	42%	11%	57%	29%	-62%
	110	51%	25%	18%	-34%	43%	39%	33%	26%	-16%	19%	18%	45%	-11%	63%	22%	-58%

Table S5 – Percent difference of freely dissolved PCB_i between treatments in SPME samples (ΔPCB_i). Values shown are the percent differences between PCB_i's geometric mean in the treatments listed in the column heading (1-[GeomeanTreatment1/GeomeanTreament2]). Positive values (blue) indicate a lower amount of PCB_i mass accumulated in SPME of the first treatment, relative to the second, at each respective timepoint. Negative values (red) indicate higher PCB_i mass accumulated. LB400 was effective at reducing freely dissolved concentrations of di- and tri-chlorinated congeners. Presence of saponin contributed to an enhanced reduction of approximately 20% throughout the incubation period, relative to the saponin-free treatment. Conditional color formatting uses maximum value of 100%, minimum of -100%, and midpoint of 0%.

SPME

		ΔPCB_i between LB400 and Control				ΔPCB_i between LB400+Sap. and Control				ΔPCB_i between LB400+Sap. and LB400				ΔPCB_i between Sap and Control			
	PCB_i	3d	11d	16d	35d	3d	11d	16d	35d	3d	11d	16d	35d	3d	11d	16d	35d
**Mono-**	1	80%	-111%	-111%	4%	88%	9%	-24%	-26%	42%	-93%	41%	-32%	1%	-58%	-34%	3%
**Di-**	4	92%	42%	83%	-33%	97%	60%	91%	63%	75%	77%	46%	73%	-8%	23%	-9%	-28%
	8	90%	29%	-205%	-1644%	96%	54%	21%	-181%	66%	67%	74%	84%	7%	-38%	60%	3%
	10	47%	-92%	46%	-181%	37%	43%	64%	-68%	-61%	-10%	32%	40%	4%	16%	-6%	-29%
**Tri-**	17	84%	75%	84%	42%	82%	25%	88%	72%	2%	82%	26%	52%	14%	35%	18%	-63%
	18+30	78%	68%	77%	-1%	76%	29%	86%	45%	6%	78%	37%	46%	11%	33%	28%	-51%
	19	-5%	6%	49%	23%	-71%	14%	58%	31%	-200%	19%	18%	11%	7%	35%	10%	-18%
	20+28	30%	34%	53%	-40%	34%	27%	64%	10%	5%	52%	24%	36%	16%	40%	45%	-98%
	27	72%	73%	79%	57%	60%	-40%	82%	65%	-44%	63%	16%	18%	19%	36%	22%	-47%
	31	81%	79%	81%	-142%	84%	36%	88%	-9%	28%	87%	35%	55%	17%	36%	41%	-148%
	32	35%	30%	45%	6%	24%	-18%	64%	22%	-6%	18%	36%	18%	13%	38%	17%	-43%
**Tetra-**	40+71	-10%	11%	53%	-8%	-5%	-7%	67%	14%	-39%	5%	31%	21%	19%	42%	38%	-95%
	42	14%	32%	76%	26%	14%	-5%	78%	42%	-114%	28%	10%	21%	27%	42%	43%	-57%
	44+47+65	-22%	26%	70%	11%	-19%	-5%	70%	28%	-145%	22%	-2%	19%	19%	42%	37%	-60%
	49+69	4%	44%	69%	22%	10%	-16%	75%	42%	-78%	35%	17%	26%	25%	44%	41%	-60%
	50+53	32%	46%	56%	13%	39%	8%	62%	27%	4%	50%	15%	16%	22%	41%	35%	-54%
	52	-9%	57%	71%	34%	0%	5%	83%	48%	-94%	59%	40%	20%	19%	42%	44%	-27%
	56	48%	-17%	-58%	-35%	29%	24%	-25%	8%	70%	12%	21%	32%	28%	41%	47%	-132%
**Penta-**	61+70+74+76	52%	4%	-3%	-132%	46%	13%	12%	-39%	63%	17%	15%	40%	29%	44%	50%	-183%
	64	35%	-3%	39%	-3%	42%	7%	57%	17%	50%	4%	31%	19%	26%	38%	47%	-40%
	66	61%	-13%	-570%	-472%	48%	21%	-471%	-296%	79%	10%	15%	31%	33%	46%	-125%	-877%
	90+101+113	49%	-7%	33%	4%	18%	28%	36%	30%	29%	23%	5%	27%	26%	46%	42%	-97%
	95	49%	10%	14%	-7%	25%	6%	34%	17%	47%	16%	23%	22%	24%	45%	43%	-80%
	110	7%	1%	39%	-11%	-34%	24%	41%	18%	-45%	26%	3%	26%	67%	43%	42%	-102%

S6. PCB Reactive Transport Modeling

A mass balance of the most abundant individual congeners (PCB_i) is solved for their concentration in the water (aqueous phase), concentration in the air, mass the SPME fiber, and mass on the PUF passive sampler using the equations denoted with an asterisk and their dependent equations shown below. The sum of PCB_i's mass in each compartment is equal to its total mass in the bioreactor system. Each equation given is followed by their dependent equations. All terms are defined or referenced in **Table S6**. The model itself ("R" code)[4] and the underlying data[5] used to produce the simulated results are both available for open-access reuse with no user registration or cost requirements at the Iowa Research Online (IRO) data repository.

S6.1. Mass-Balance Equations for Reactive Transport Model

$$V_w \frac{dC_w}{dt} = -k_a C_w V_w + k_d C_p V_{pw} - A_{aw} k_{aw} \left(C_w - {C_a}/{K_{aw}} \right) - k_b C_w V_w$$ **Equation 1***

$$\rightarrow C_{w_i} = \frac{C_t d_s}{1 + MK}$$ **Equation 2**

$$\rightarrow K = f_{oc} K_{oc}$$ **Equation 3**

$$\rightarrow K_{oc} = 10^{\alpha log K_{ow} + \beta}$$ **Equation 4**

$$\rightarrow C_p = MKC_w$$ **Equation 5**

$$\rightarrow k_{aw} = \left(\frac{1}{K_{aw}^{\text{air}} K_{aw}^{T}} + \frac{1}{K_{aw}^{\text{H}_2\text{O}}} \right)^{-1}$$ **Equation 6**

$$\rightarrow K_{aw}^{T} = K_{aw} e^{\left(\frac{-\Delta U_{ow}}{R} \left(\frac{1}{T} - \frac{1}{T_{\text{std}}} \right) \right)}$$ **Equation 7**

$$\rightarrow K_{aw}^{\text{air}} = V_{\text{H}_2\text{O - air}} \left(\frac{D_{\text{PCB}_i\text{ - air}}}{D_{\text{H}_2\text{O - air}}} \right)^{0.67}$$ **Equation 8**

$$\rightarrow D_{\text{PCB}_i\text{ - air}} = D_{\text{H}_2\text{O - air}} \sqrt{\frac{MW_{\text{PCB}_i}}{MW_{\text{H}_2\text{O}}}}$$ **Equation 9**

$$\rightarrow D_{\text{PCB}_i\text{ - H}_2\text{O}} = D_{CO_2\text{ - H}_2\text{O}} \sqrt{\frac{MW_{\text{PCB}_i}}{MW_{CO_2}}}$$ **Equation 10**

$$\rightarrow K_{aw}^{\text{H}_2\text{O}} = V_{CO_2\text{ - H}_2\text{O}} \sqrt{\left(\frac{Sc_{\text{PCB}_i}}{Sc_{CO_2}} \right)}$$ **Equation 11**

$$\rightarrow Sc_{\text{PCB}_i} = {V_{\text{H}_2\text{O}}}/{D_{\text{PCB}_i\text{ - H}_2\text{O}}}$$ **Equation 12**

$$V_a \frac{dC_a}{dt} = A_{aw} k_{aw} \left(C_w - \frac{C_a}{K_{aw}} \right)$$ **Equation 13***

S6.2. Passive Sampler Mass-Balance Equations for Reactive Transport Model

$$\frac{dm_f}{dt} = \frac{k_o A_f}{L} \left(C_w - \frac{m_f}{V_f K_f} \right)$$ **Equation 14***

$$\rightarrow K_f = 10^{1.06(logK_{ow} - 1.16)}$$ **Equation 15**

$$\frac{dm_{\text{PUF}}}{dt} = r\left(C_a - \frac{m_{\text{PUF}}}{V_{\text{PUF}} K_{\text{PUF}} D}\right)$$ **Equation 16***

$$\rightarrow K_{puf} = 10^{0.6366(logK_{oa} - 3.1774)}$$ **Equation 17**

S6.3. Definitions of Terms Used in Reactive Transport Model Equations

Table S6 – Definitions of each term used in the mass-balance equations for the reactive transport model and their dependent equations. Each symbol is described in the order it appears in the equations above, followed by its dependent terms or equations. If a term has a constant value, it is given. If not, the appropriate equation that can be used to determine its value is referenced in the "Value" column. An asterisk denotes equations that are part of the system of ordinary differential equations solved simultaneously in the model. If applicable, a reference is given in the "Reference" column.

Symbol	Description	Value	Units	Reference
V_w	Volume of aqueous solution in bioreactor	10×10^{-5}	m^3	This study
C_w	PCB_i concentration in aqueous phase	**Equation 1***	ng/L	
k_a	PCB_i absorption rate	Congener- specific, fitted parameter	day^{-1}	
C_{w_i}	PCB_i concentration in water initially	**Equation 2**	ng/L	
C_t	PCB_i concentration in sediment	Congener-specific	ng/g	(Dataset)
d_s	Density of sediment	900	g/L	
M	Ratio of Sediment to aqueous solution in bioreactor	0.1	kg/L	This study
K	PCB_i particle-aqueous phase partition coefficient	**Equation 3**	L/kg	
f_{oc}	Fraction of organic carbon in sediment particle	0.03	kg-oc/kg	This study
K_{oc}	PCB_i organic carbon partition coefficient	**Equation 4**	L/kg	(Nguyen et. al., 2005)[6]
α	Empirical constant obtained via linear regression	Congener & sediment-specific		(Nguyen et. al., 2005)[6]
K_{ow}	PCB_i octanol-water partition coefficient	Congener-specific	L/kg	(Hawker & Cornell, 1988)[7]
β	Empirical constant obtained via linear regression	Congener & sediment-specific		(Nguyen et. al., 2005)[6]
k_d	PCB_i desorption rate	Congener- specific, fitted parameter	day^{-1}	
C_p	Sediment particle concentration in aqueous phase	**Equation 5**	kg/L	

V_{pw}	Volume of porewater in bioreactor	2.5×10^{-6}	m^3	This study
A_{aw}	Area of air-aqueous phase interface in bioreactor	30	cm^2	This study
k_{aw}	PCB_i air-aqueous phase mass transfer coefficient	**Equation 6**	cm/day	(Martinez et. al., 2010)[8]
K_{aw}^{T}	PCB_i Dimensionless Henry's Law constant at temperature, T	**Equation 7**		(Goss, 2006)[9]
K_{aw}	PCB_i dimensionless Henry's Law constant at standard temperature, T_{std}	0.0130452		(Dunnivant et. al., 1992)[10]
ΔU_{ow}	PCB_i octanol-water internal energy of transfer	55517.96	J/mol	(Li et. al., 2003)[11]
R	Molar gas constant	8.3144	J/mol-K	
T	Water temperature	293.15	K	This study
T_{std}	Standard temperature for air and water	298.15	K	
K_{aw}^{air}	PCB_i air-side mass transfer coefficient	**Equation 8**	m/s	(Martinez et. al., 2010)[8]
$V_{H_2O\text{ - air}}$	H_2O's air-side mass transfer coefficient without ventilation	0.003	m/s	(Comenges et. al., 2017)[12]
$D_{PCB_i\text{ - air}}$	PCB_i gas-phase diffusion coefficient at T_{std} and atmospheric pressure, P_{atm} = 1,013.25 mbars	**Equation 9**	cm^2/s	(Martinez et. al., 2010)[8]
$D_{H_2O\text{ - air}}$	Water gas-phase diffusion coefficient at T_{std} and P_{atm}	0.2743615	cm^2/s	(Martinez et. al., 2010)[8]
MW_{PCB_i}	PCB_i molecular weight	Congener-specific	g/mol	
MW_{H_2O}	H_2O molecular weight	18.0152	g/mol	
$D_{PCB_i\text{ - }H_2O}$	PCB_i diffusion coefficient in water at T_{std} and P_{atm}	**Equation 10**	cm^2/s	(Martinez et. al., 2010)[8]
$D_{CO_2\text{ - }H_2O}$	CO_2 diffusion coefficient in water at T_{std} and P_{atm}	1.67606×10^{-5}	cm^2/s	(Martinez et. al., 2010)[8]
MW_{CO_2}	CO_2 molecular weight	44.0094	g/mol	
$K_{aw}^{H_2O}$	PCB_i water-side mass transfer coefficient	**Equation 11**	m/s	(Martinez et. al., 2010)[8]
$V_{CO_2\text{ - }H_2O}$	CO_2's water-side mass transfer coefficient without ventilation	0.041	m/s	(Comenges et. al., 2017)[12]
Sc_{CO_2}	CO_2 Schmidt number at T_{std} and P_{atm}	600		(Comenges et. al., 2017)[12]
Sc_{PCB_i}	PCB_i Schmidt number at T_{std} and P_{atm}	**Equation 12**		(Martinez et. al., 2010)[8]
V_{H_2O}	Kinematic viscosity of water T_{std} and P_{atm}	0.010072884	cm^2/s	(Martinez et. al., 2010)[8]
C_a	PCB_i concentration in air	**Equation 13**	ng/m^3	

k_b	PCB_i biotransformation rate by LB400	Congener-specific	day^{-1}	(Bako et. al., 2021ab)[13,14]
V_a	Volume of air in headspace of bioreactor	1.25×10^{-5}	m^3	This study
m_f	PCB_i mass in SPME fiber	**Equation 14***	ng/cm	
k_o	PCB_i fiber-aqueous phase mass transfer coefficient	Congener-specific	cm/day	
A_f	Average SPME fiber surface area	0.138	cm^2	This study
L	Average SPME fiber length	20	cm	This study
V_f	Volume of PDMS coating on fiber	6.9×10^{-8}	L/cm	This study
K_f	PCB_i fiber-aqueous phase partition coefficient	**Equation 15**		(Lu et. al., 2011)[15]
m_{PUF}	PCB_i mass in PUF	**Equation 16***	ng	
r	PUF sampling rate	0.0045	m^3/day	This study
V_{puf}	PUF Volume	0.000029	m^3	This study
D	PUF density	21,300	g/m^3	This study
K_{puf}	PUF-air partition coefficient	**Equation 17**	m^3/g	(Shoeib & Harner, 2002)[16]
K_{oa}	PCB_i octanol-air partition coefficient	Congener-specific		(Harner & Bidleman, 1996)[17]

S6.4. Results of PCB Reactive Transport Model for Most Abundant PCB Congeners

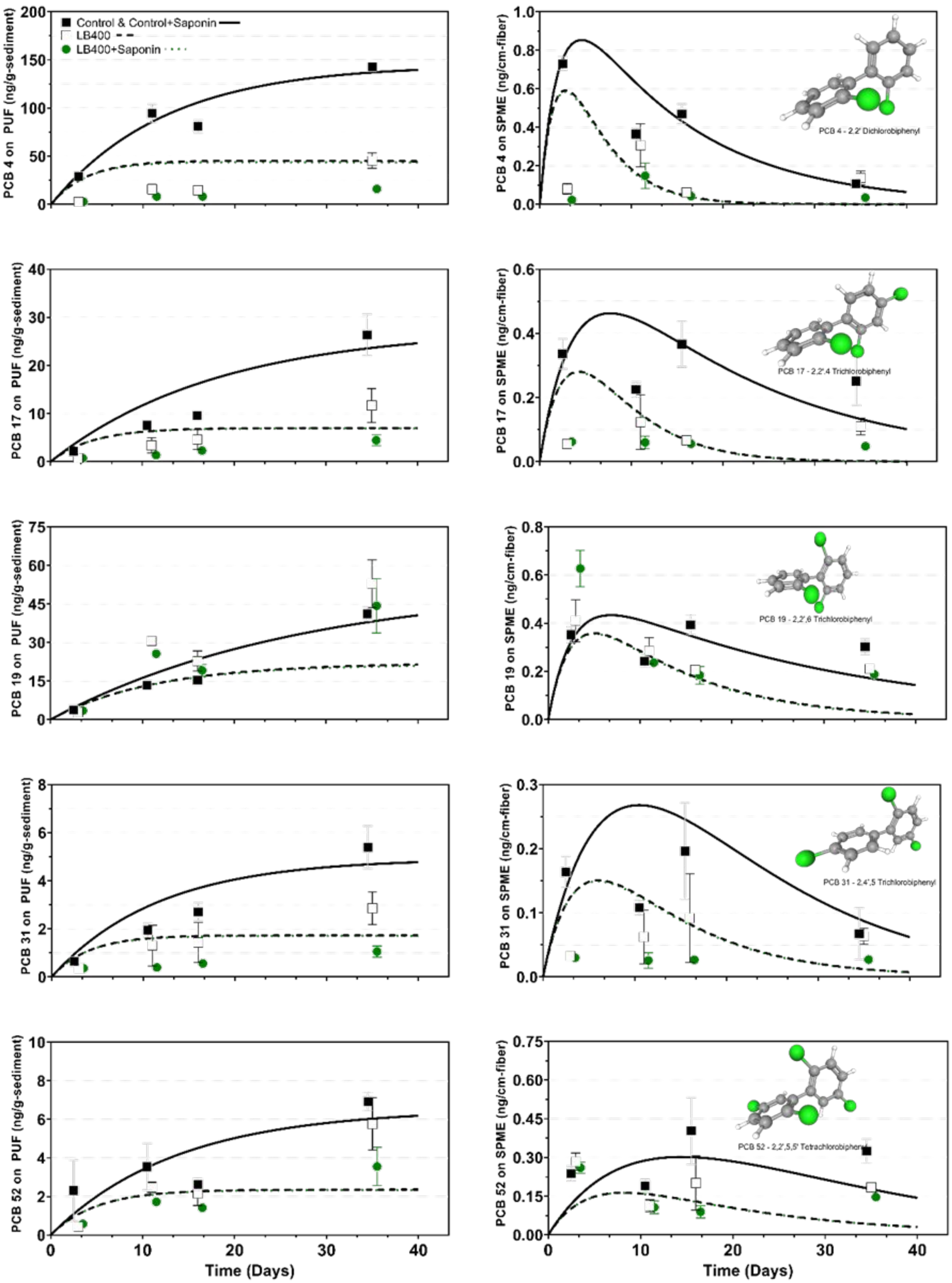

Figure S5 – Accumulation of the most abundant individual congeners on passive samplers deployed in bioreactors, over 35 days with chemical structures inset. PUF samples are shown on the left and SPME on the right. Symbols indicate experimental results, and the corresponding dotted lines represent results of the PCB reactive transport model. Error bars represent standard error of triplicate measurements in non-bioaugmented controls (n = 3) and quadruplet in bioaugmented treatments (n = 4).

S7. Statistical Analyses

S7.1. Summary of Results for LC-PCB Statistical Analyses

A three-way mixed effect analysis was carried out to determine the effects of time, saponin, and LB400 on LC-PCB accumulation in both PUF and SPME passive samplers. The fixed effects were fitted to a restricted maximum likelihood (REML) linear mixed effects model in GraphPad Prism™. Residual analysis was performed to test for the assumptions of the three-way mixed effect analysis (**Figure S6**). Additionally, normality and homogeneity of variances were assessed using Shapiro-Wilk's and Levene's tests, respectively, using R software. Residuals were normally distributed ($p > 0.05$) and there was homogeneity of variances ($p > 0.05$). Statistical significance was accepted at the $p < 0.05$ level for simple main effects.

For PUF samples, there was a significant interaction effect detected between time and bioaugmentation, $F(3, 16) = 4.470$, $p = 0.0184$. In addition, bioaugmentation, $F(1, 16) = 161.6$, and time, $F(3, 23) = 74.18$, were highly statistically significant main effects ($p < 0.0001$) whereas saponin was not, $F(1, 23) = 3.006$, $p = 0.0964$ (**Table S7**).

For SPME samples, there was a significant interaction effect detected between time and bioaugmentation, $F(3, 16) = 4.445$, $p = 0.0187$. Time and bioaugmentation were significant main effects $F(3, 24) = 3.329$, $p = 0.0365$, and $F(1, 16) = 32.39$, $p < 0.0001$ whereas saponin was not, $F(1, 23) = 3.006$, $p = 0.0964$ (**Table S13**).

Because saponin was an insignificant main effect in both types of samplers, a two-way mixed effects analysis using the residual error of the three-way mixed effects model was run using data which consolidated bioaugmented groups (Control and Control+Saponin) and non-bioaugmented groups (LB400 and LB400+Saponin). Results of this analysis found that bioaugmentation had a highly significant main effect on ΣPCB accumulation in both PUF (**Table S8**) and SPME (**Table S14**; $p < 0.005$)

All simple pairwise comparisons between treatments at the same timepoint were made using a Holm-Šídák-adjusted post-hoc t-test. Results of these comparisons can be observed in **Table S9-Table S12** and **Table S15-Table S18** for PUF and SPME, respectively.

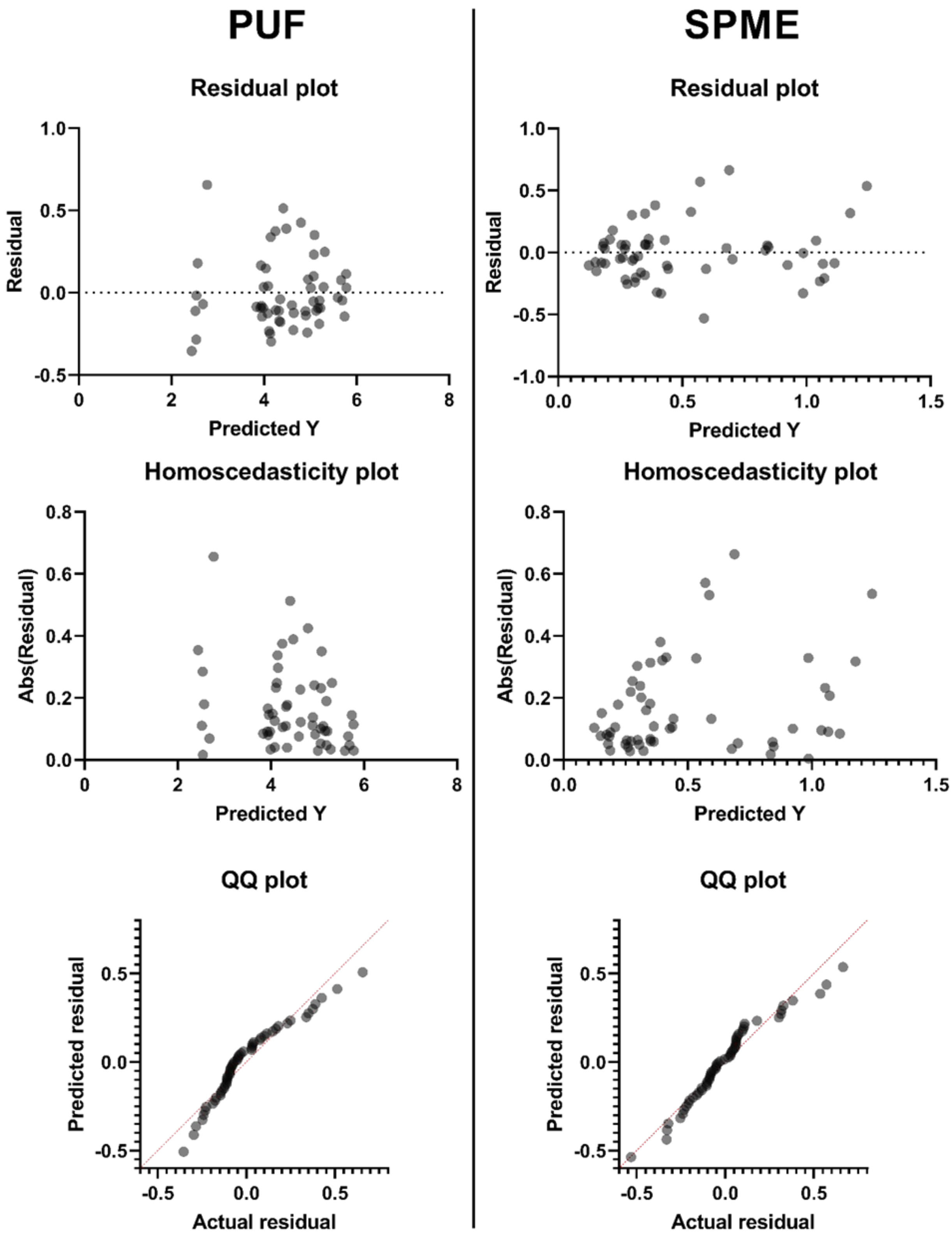

Figure S6 – Residual, homoscedasticity, and QQ plots for results of three-way mixed-effect analyses of PUF samples (left) and SPME samples (right).

S7.2. *Tabular Results for Statistical Analysis on PUF Measurements*

Below are tables generated from the three-way and two-way mixed effect analyses conducted on log10-transformed PUF measurements in GraphPad Prism™.

Table S7 – Tabular results of three-way mixed effects analysis on log-transformed LC-PCB PUF measurements using LB400, saponin, and time as fixed effects fitted to a restricted maximum likelihood (REML) linear mixed effects model with sphericity assumed. α = 0.05.

Fixed effects (type III)	P value	P value summary	Statistically significant (P < 0.05)?	F (DFn, DFd)
Time	<0.0001	****	Yes	F (3, 23) = 74.18
Bioaugmentation	<0.0001	****	Yes	F (1, 16) = 161.6
Saponin	0.0964	ns	No	F (1, 23) = 3.006
Time x Bioaugmentation	0.0184	*	Yes	F (3, 16) = 4.470
Time x Saponin	0.9821	ns	No	F (3, 23) = 0.05603
Bioaugmentation x Saponin	0.1014	ns	No	F (1, 16) = 3.020
Time x Bioaugmentation x Saponin	0.2595	ns	No	F (3, 16) = 1.473

Table S8 – Tabular results of two-way mixed effects analysis on consolidated LC-PCB PUF data using LB400 and time as fixed effects fitted to a restricted maximum likelihood (REML) linear mixed effects model with no sphericity assumed (Geisser-Greenhouse Correction applied). α = 0.05.

Fixed effects (type III)	P value	P value summary	Statistically significant (P < 0.05)?	F (DFn, DFd)	Geisser-Greenhouse's epsilon
Time	<0.0001	****	Yes	F (2.366, 27.61) = 121.7	0.7887
Treatment	<0.0001	****	Yes	F (1, 12) = 63.35	
Time x Treatment	0.0111	*	Yes	F (3, 35) = 4.291	

Table S9 – Results of Holm-Šídák's multiple comparisons tests following three-way mixed effects analysis on log-transformed LC-PCB PUF measurements.

Holm-Šídák's multiple comparisons test	Predicted (LS) mean diff.	Below threshold?	Summary	Adjusted P Value
3d:Control vs. 3d:Saponin	0.1807	No	ns	>0.9999
3d:Control vs. 3d:LB400	1.565	Yes	***	0.0003
3d:Control vs. 3d:LB400 + Sap.	1.583	Yes	****	<0.0001
3d:Saponin vs. 3d:LB400	1.384	Yes	***	0.0004
3d:Saponin vs. 3d:LB400 + Sap.	1.402	Yes	***	0.0007
3d:LB400 vs. 3d:LB400 + Sap.	0.01817	No	ns	>0.9999
11d:Control vs. 11d:Saponin	0.04004	No	ns	>0.9999
11d:Control vs. 11d:LB400	0.718	No	ns	0.2119
11d:Control vs. 11d:LB400 + Sap.	1.021	Yes	*	0.0113
11d:Saponin vs. 11d:LB400	0.678	No	ns	0.3311
11d:Saponin vs. 11d:LB400 + Sap.	0.9813	Yes	*	0.0238
11d:LB400 vs. 11d:LB400 + Sap.	0.3033	No	ns	0.9966
16d:Control vs. 16d:Saponin	0.08345	No	ns	>0.9999
16d:Control vs. 16d:LB400	0.7531	No	ns	0.1644
16d:Control vs. 16d:LB400 + Sap.	1.055	Yes	**	0.0077
16d:LB400 vs. 16d:LB400 + Sap.	0.3022	No	ns	0.9966
35d:Control vs. 35d:Saponin	-0.1796	No	ns	>0.9999
35d:Control vs. 35d:LB400	0.3779	No	ns	0.9662
35d:Control vs. 35d:LB400 + Sap.	0.9602	Yes	*	0.0224
35d:Saponin vs. 35d:LB400	0.5575	No	ns	0.6731
35d:Saponin vs. 35d:LB400 + Sap.	1.14	Yes	**	0.0064
35d:LB400 vs. 35d:LB400 + Sap.	0.5822	No	ns	0.4495

Table S10 – Details of Holm-Šídák's multiple comparisons tests following three-way mixed effects analysis on log-transformed LC-PCB PUF measurements.

Test details	Predicted (LS) mean 1	Predicted (LS) mean 2	Predicted (LS) mean diff.	SE of diff.	N1	N2	t	DF
3d:Control vs. 3d:Saponin	4.154	3.974	0.1807	0.2617	3	3	0.6904	39
3d:Control vs. 3d:LB400	4.154	2.59	1.565	0.2298	3	3	6.808	16
3d:Control vs. 3d:LB400 + Sap.	4.154	2.572	1.583	0.2456	3	4	6.443	39
3d:Saponin vs. 3d:LB400	3.974	2.59	1.384	0.2617	3	3	5.288	39
3d:Saponin vs. 3d:LB400 + Sap.	3.974	2.572	1.402	0.2185	3	4	6.415	16
3d:LB400 vs. 3d:LB400 + Sap.	2.59	2.572	0.01817	0.2456	3	4	0.07396	39
11d:Control vs. 11d:Saponin	5.095	5.055	0.04004	0.2608	3	3	0.1535	39
11d:Control vs. 11d:LB400	5.095	4.377	0.718	0.2185	3	4	3.286	16
11d:Control vs. 11d:LB400 + Sap.	5.095	4.074	1.021	0.2447	3	4	4.174	39
11d:Saponin vs. 11d:LB400	5.055	4.377	0.678	0.2447	3	4	2.771	39
11d:Saponin vs. 11d:LB400 + Sap.	5.055	4.074	0.9813	0.2185	3	4	4.49	16
11d:LB400 vs. 11d:LB400 + Sap.	4.377	4.074	0.3033	0.2274	4	4	1.334	39
16d:Control vs. 16d:Saponin	5.039	4.956	0.08345	0.2608	3	3	0.32	39
16d:Control vs. 16d:LB400	5.039	4.286	0.7531	0.2185	3	4	3.446	16
16d:Control vs. 16d:LB400 + Sap.	5.039	3.984	1.055	0.2447	3	4	4.313	39
16d:LB400 vs. 16d:LB400 + Sap.	4.286	3.984	0.3022	0.2274	4	4	1.329	39
35d:Control vs. 35d:Saponin	5.629	5.808	-0.1796	0.2608	3	3	0.6885	39
35d:Control vs. 35d:LB400	5.629	5.251	0.3779	0.2185	3	4	1.729	16
35d:Control vs. 35d:LB400 + Sap.	5.629	4.669	0.9602	0.2447	3	4	3.924	39
35d:Saponin vs. 35d:LB400	5.808	5.251	0.5575	0.2447	3	4	2.278	39
35d:Saponin vs. 35d:LB400 + Sap.	5.808	4.669	1.14	0.2185	3	4	5.215	16
35d:LB400 vs. 35d:LB400 + Sap.	5.251	4.669	0.5822	0.2274	4	4	2.56	39

Table S11 – Results of Holm-Šídák's multiple comparisons tests following two-way mixed effects analysis on consolidated LC-PCB PUF measurements.

Holm-Šídák's multiple comparisons test<br>Non-bioaugmented - Bioaugmented	Mean Diff.	Below threshold?	Summary	Adjusted P Value
3d	1.451	Yes	***	0.0001
11d	0.8563	Yes	***	0.0003
16d	0.8662	Yes	***	0.0003
35d	0.7468	Yes	**	0.0013

Table S12 – Details of Holm-Šídák's multiple comparisons tests following two-way mixed effects analysis on consolidated LC-PCB PUF measurements.

Test details<br>Non-bioaugmented - Bioaugmented	Mean 1	Mean 2	Mean Diff.	SE of diff.	N1	N2	t	DF
3d	4.031	2.579	1.451	0.2081	6	7	6.974	10.72
11d	5.082	4.226	0.8563	0.1559	6	8	5.492	12
16d	5.001	4.135	0.8662	0.1499	6	8	5.777	11.39
35d	5.707	4.96	0.7468	0.1598	6	8	4.674	8.603

S7.3. Tabular Results for Statistical Analysis on SPME Measurements

Below are tables generated from three-way and two-way mixed effect analyses conducted on log10-transformed SPME measurements in GraphPad Prism™.

Table S13 – Tabular results of three-way mixed effects analysis on log-transformed LC-PCB SPME measurements using LB400, saponin, and time as fixed effects fitted to a restricted maximum likelihood (REML) linear mixed effects model with sphericity assumed. α = 0.05.

Fixed effects (type III)	P value	P value summary	Statistically significant (P < 0.05)?	F (DFn, DFd)
Time	0.0365	*	Yes	F (3, 24) = 3.329
Bioaugmented	<0.0001	****	Yes	F (1, 16) = 32.39
Saponin	0.6282	ns	No	F (1, 24) = 0.2406
Time x Bioaugmented	0.0187	*	Yes	F (3, 16) = 4.445
Time x Saponin	0.149	ns	No	F (3, 24) = 1.947
Bioaugmented x Saponin	0.941	ns	No	F (1, 16) = 0.005646
Time x Bioaugmented x Saponin	0.5614	ns	No	F (3, 16) = 0.7076

Table S14 – Tabular results of two-way mixed effects analysis on consolidated LC-PCB SPME data using LB400 and time as fixed effects fitted to a restricted maximum likelihood (REML) linear mixed effects model with no sphericity assumed (Geisser-Greenhouse Correction applied). α = 0.05.

Fixed effects (type III)	P value	P value summary	Statistically significant (P < 0.05)?	F (DFn, DFd)	Geisser-Greenhouse's epsilon
Time	0.0219	*	Yes	F (2.447, 29.36) = 4.028	0.8156
Bioaugmented	0.0004	***	Yes	F (1, 12) = 23.50	
Time x Bioaugmented	0.0127	*	Yes	F (3, 36) = 4.144	

Table S15 – Results of Holm-Šídák's multiple comparisons tests following three-way mixed effects analysis on log-transformed LC-PCB SPME measurements.

Holm-Šídák's multiple comparisons test	Predicted (LS) mean diff.	Below threshold?	Summary	Adjusted P Value
3d:Control vs. 3d:Saponin	0.08196	No	ns	>0.9999
3d:Control vs. 3d:LB400	0.9047	No	ns	0.0904
3d:Control vs. 3d:LB400 + Sap.	0.7985	No	ns	0.1634
3d:Saponin vs. 3d:LB400	0.8228	No	ns	0.1258
3d:Saponin vs. 3d:LB400 + Sap.	0.7166	No	ns	0.3939
3d:LB400 vs. 3d:LB400 + Sap.	-0.1062	No	ns	>0.9999
11d:Control vs. 11d:Saponin	0.3509	No	ns	>0.9999
11d:Control vs. 11d:LB400	0.2639	No	ns	>0.9999
11d:Control vs. 11d:LB400 + Sap.	0.5602	No	ns	0.8591
11d:Saponin vs. 11d:LB400	-0.087	No	ns	>0.9999
11d:Saponin vs. 11d:LB400 + Sap.	0.2092	No	ns	>0.9999
11d:LB400 vs. 11d:LB400 + Sap.	0.2962	No	ns	>0.9999
16d:Control vs. 16d:Saponin	0.2152	No	ns	>0.9999
16d:Control vs. 16d:LB400	0.7084	No	ns	0.4148
16d:Control vs. 16d:LB400 + Sap.	0.7487	No	ns	0.2597
16d:LB400 vs. 16d:LB400 + Sap.	0.04032	No	ns	>0.9999
35d:Control vs. 35d:Saponin	-0.4506	No	ns	0.998
35d:Control vs. 35d:LB400	-0.08042	No	ns	>0.9999
35d:Control vs. 35d:LB400 + Sap.	-0.1601	No	ns	>0.9999
35d:Saponin vs. 35d:LB400	0.3702	No	ns	0.9999
35d:Saponin vs. 35d:LB400 + Sap.	0.2906	No	ns	>0.9999
35d:LB400 vs. 35d:LB400 + Sap.	-0.07965	No	ns	>0.9999

Table S16 – Details of Holm-Šídák's multiple comparisons tests following three-way mixed effects analysis on log-transformed LC-PCB SPME measurements.

Test details	Predicted (LS) mean 1	Predicted (LS) mean 2	Predicted (LS) mean diff.	SE of diff.	N1	N2	t	DF
3d:Control vs. 3d:Saponin	1.109	1.027	0.08196	0.2521	3	3	0.3251	40
3d:Control vs. 3d:LB400	1.109	0.2042	0.9047	0.2203	3	4	4.107	16
3d:Control vs. 3d:LB400 + Sap.	1.109	0.3104	0.7985	0.2361	3	4	3.382	40
3d:Saponin vs. 3d:LB400	1.027	0.2042	0.8228	0.2361	3	4	3.484	40
3d:Saponin vs. 3d:LB400 + Sap.	1.027	0.3104	0.7166	0.2203	3	4	3.253	16
3d:LB400 vs. 3d:LB400 + Sap.	0.2042	0.3104	-0.1062	0.219	4	4	0.485	40
11d:Control vs. 11d:Saponin	0.7305	0.3796	0.3509	0.2521	3	3	1.392	40
11d:Control vs. 11d:LB400	0.7305	0.4666	0.2639	0.2203	3	4	1.198	16
11d:Control vs. 11d:LB400 + Sap.	0.7305	0.1704	0.5602	0.2361	3	4	2.372	40
11d:Saponin vs. 11d:LB400	0.3796	0.4666	-0.087	0.2361	3	4	0.3684	40
11d:Saponin vs. 11d:LB400 + Sap.	0.3796	0.1704	0.2092	0.2203	3	4	0.9497	16
11d:LB400 vs. 11d:LB400 + Sap.	0.4666	0.1704	0.2962	0.219	4	4	1.353	40
16d:Control vs. 16d:Saponin	1.09	0.8743	0.2152	0.2521	3	3	0.8537	40
16d:Control vs. 16d:LB400	1.09	0.3811	0.7084	0.2203	3	4	3.216	16
16d:Control vs. 16d:LB400 + Sap.	1.09	0.3408	0.7487	0.2361	3	4	3.171	40
16d:LB400 vs. 16d:LB400 + Sap.	0.3811	0.3408	0.04032	0.219	4	4	0.1842	40
35d:Control vs. 35d:Saponin	0.1774	0.628	-0.4506	0.2521	3	3	1.787	40
35d:Control vs. 35d:LB400	0.1774	0.2578	-0.08042	0.2203	3	4	0.365	16
35d:Control vs. 35d:LB400 + Sap.	0.1774	0.3374	-0.1601	0.2361	3	4	0.6779	40
35d:Saponin vs. 35d:LB400	0.628	0.2578	0.3702	0.2361	3	4	1.568	40
35d:Saponin vs. 35d:LB400 + Sap.	0.628	0.3374	0.2906	0.2203	3	4	1.319	16
35d:LB400 vs. 35d:LB400 + Sap.	0.2578	0.3374	-0.07965	0.219	4	4	0.3638	40

Table S17 – Results of Holm-Šídák's multiple comparisons tests following two-way mixed effects analysis on consolidated LC-PCB SPME measurements.

Holm-Šídák's multiple comparisons test Non-bioaugmented - Bioaugmented	Mean Diff.	Below threshold?	Summary	Adjusted P Value
3d	0.8152	Yes	***	0.0002
11d	0.2383	No	ns	0.3062
16d	0.6246	Yes	*	0.0331
35d	0.1046	No	ns	0.6313

Table S18 – Details of Holm-Šídák's multiple comparisons tests following two-way mixed effects analysis on consolidated SPME measurements.

Test details Non-bioaugmented - Bioaugmented	Mean 1	Mean 2	Mean Diff.	SE of diff.	N1	N2	t	DF
3d	1.072	0.2573	0.8152	0.1173	6	8	6.949	9.963
11d	0.5568	0.3185	0.2383	0.1619	6	8	1.472	11.87
16d	0.9855	0.3609	0.6246	0.1989	6	8	3.14	9.507
35d	0.4022	0.2976	0.1046	0.2065	6	8	0.5064	5.785

S8. PCB Quantification

Table S19 – PCB precursor and product masses of labeled and unlabeled calibration standards employed in multiple reaction monitoring mode on the triple quadrupole mass spectrometer.[a]

Cl Homolog	Precursor Mass	Product Mass
Monochlorinated	188	152
Dichlorinated	222	152
Trichlorinated	258	186
Tetrachlorinated	291.9	222
Pentachlorinated	325.9	255.9
Hexachlorinated	359.8	289.9
Heptachlorinated	393.8	323.9
Octachlorinated	429.7	359.8
Nonachlorinated	463.7	393.8
Decachlorinated	497.7	427.9
65D Tetrachlorinated (surrogate std.)	269.9	227

[a] Standards were from AccuStandard, New Haven, CT, USA

References

(1) Mattes, T. E.; Ewald, J. M.; Liang, Y.; Martinez, A.; Awad, A.; Richards, P.; Hornbuckle, K. C.; Schnoor, J. L. PCB Dechlorination Hotspots and Reductive Dehalogenase Genes in Sediments from a Contaminated Wastewater Lagoon. *Environ. Sci. Pollut. Res.* **2018**, *25* (17), 16376–16388. https://doi.org/10.1007/s11356-017-9872-x.

(2) Petrić, I.; Hršak, D.; Fingler, S.; Udiković-Kolić, N.; Bru, D.; Martin-Laurent, F. Insight in the PCB-Degrading Functional Community in Long-Term Contaminated Soil under Bioremediation. *J. Soils Sediments* **2011**, *11* (2), 290–300. https://doi.org/10.1007/s11368-010-0299-y.

(3) Bustin, S. A.; Benes, V.; Garson, J. A.; Hellemans, J.; Huggett, J.; Kubista, M.; Mueller, R.; Nolan, T.; Pfaffl, M. W.; Shipley, G. L.; Vandesompele, J.; Wittwer, C. T. The MIQE Guidelines: Minimum Information for Publication of Quantitative Real-Time PCR Experiments. *Clin. Chem.* **2009**, *55* (4), 611–622. https://doi.org/10.1373/clinchem.2008.112797.

(4) Bako, C. M.; Martinez, A. R Code for Polychlorinated Biphenyl (PCB) Reactive Transport Model. *Iowa Res. Online* **2022**. https://doi.org/10.25820/code.006163.

(5) Bako, C. M.; Martinez, A.; Ewald, J. M.; Hua, J. B. X.; Schnoor, J. L.; Mattes, T. E. Dataset Describing Polychlorinated Biphenyl (PCB) Congener Accumulation on Passive Samplers and Mass Transport in Sediment Slurry Bioreactors Bioaugmented with *Paraburkholderia xenovorans* LB400. *Iowa Res. Online* **2021**, *V1*. https://doi.org/10.25820/data.006160.

(6) Nguyen, T. H.; Goss, K.-U.; Ball, W. P. Polyparameter Linear Free Energy Relationships for Estimating the Equilibrium Partition of Organic Compounds between Water and the Natural Organic Matter in Soils and Sediments. *Environ. Sci. Technol.* **2005**, *39* (4), 913–924. https://doi.org/10.1021/es048839s.

(7) Hawker, D. W.; Connell, D. W. Octanol-Water Partition Coefficients of Polychlorinated Biphenyl Congeners. *Environ. Sci. Technol.* **1988**, *22* (4), 382–387. https://doi.org/10.1021/es00169a004.

(8) Martinez, A.; Wang, K.; Hornbuckle, K. C. Fate of PCB Congeners in an Industrial Harbor of Lake Michigan. *Environ. Sci. Technol.* **2010**, *44* (8), 2803–2808. https://doi.org/10.1021/es902911a.

(9) Goss, K.-U. Prediction of the Temperature Dependency of Henry's Law Constant Using Poly-Parameter Linear Free Energy Relationships. *Chemosphere* **2006**, *64* (8), 1369–1374. https://doi.org/10.1016/j.chemosphere.2005.12.049.

(10) Dunnivant, F. M.; Elzerman, A. W.; Jure, P. C.; Hasan, M. N. Quantitative Structure-Property Relationships for Aqueous Solubilities and Henry's Law Constants of Polychlorinated Biphenyls. *Environ. Sci. Technol.* **1992**, *26* (8), 1567–1573. https://doi.org/10.1021/es00032a012.

(11) Li, N.; Wania, F.; Lei, Y. D.; Daly, G. L. A Comprehensive and Critical Compilation, Evaluation, and Selection of Physical–Chemical Property Data for Selected Polychlorinated Biphenyls. *J. Phys. Chem. Ref. Data* **2003**, *32* (4), 1545–1590. https://doi.org/10.1063/1.1562632.

(12) Comenges, J. M. Z.; Joossens, E.; Benito, J. V. S.; Worth, A.; Paini, A. Theoretical and Mathematical Foundation of the Virtual Cell Based Assay – A Review. *Toxicol. Vitr.* **2017**, *45*, 209–221. https://doi.org/10.1016/j.tiv.2016.07.013.

(13) Bako, C. M.; Mattes, T. E.; Marek, R. F.; Hornbuckle, K. C.; Schnoor, J. L. Biodegradation of PCB Congeners by *Paraburkholderia xenovorans* LB400 in Presence and Absence of Sediment during Lab Bioreactor Experiments. *Environ. Pollut.* **2021**, *271*, 116364. https://doi.org/10.1016/j.envpol.2020.116364.

(14) Bako, C. M.; Mattes, T. E.; Marek, R. F.; Hornbuckle, K. C.; Schnoor, J. L. Dataset Describing Biodegradation of Individual Polychlorinated Biphenyl Congeners (PCBs) by *Paraburkholderia xenovorans* LB400 in Presence and Absence of Sediment Slurry. *Data Br.* **2021**, *35*, 106821. https://doi.org/10.1016/j.dib.2021.106821.

(15) Lu, X.; Skwarski, A.; Drake, B.; Reible, D. D. Predicting Bioavailability of PAHs and PCBs with Porewater

Concentrations Measured by Solid-Phase Microextraction Fibers. *Environ. Toxicol. Chem.* **2011**, *30* (5), 1109–1116. https://doi.org/10.1002/etc.495.

(16) Shoeib, M.; Harner, T. Characterization and Comparison of Three Passive Air Samplers for Persistent Organic Pollutants. *Environ. Sci. Technol.* **2002**, *36* (19), 4142–4151. https://doi.org/10.1021/es020635t.

(17) Harner, T.; Bidleman, T. F. Measurements of Octanol−Air Partition Coefficients for Polychlorinated Biphenyls. *J. Chem. Eng. Data* **1996**, *41* (4), 895–899. https://doi.org/10.1021/je960097y.
